# Overexpression of a Novel ERF-X-Type Transcription Factor, *OsERF106MZ*, Reduces Shoot Growth and Tolerance to Salinity Stress in Rice

**DOI:** 10.1186/s12284-021-00525-5

**Published:** 2021-09-20

**Authors:** Hung-Chi Chen, Tzu-Cheng Chien, Tsung-Yang Chen, Ming-Hau Chiang, Ming-Hsin Lai, Men-Chi Chang

**Affiliations:** 1 Department of Agronomy, National Taiwan University, No. 1, Section 4, Roosevelt Road, Taipei, Taiwan, ROC; 2 Crop Science Division, Taiwan Agricultural Research Institute, Taichung, Taiwan

**Keywords:** AP2/ERF, Ion homeostasis, Salinity stress, Shoot growth, Transcription factor

## Abstract

Transcription factors (TFs) such as ethylene-responsive factors (ERFs) are important for regulating plant growth, development, and responses to abiotic stress. Notably, more than half of the rice ERF-X group members, including *ethylene-responsive factor 106* (*OsERF106*), are abiotic stress-responsive genes. However, their regulatory roles in abiotic stress responses remain poorly understood. *OsERF106*, a salinity-induced gene of unknown function, is annotated differently in RAP-DB and MSU RGAP. In this study, we isolated a novel (i.e., previously unannotated) *OsERF106* gene, designated *OsERF106MZ* (GenBank accession No. MZ561461), and investigated its role in regulating growth and the response to salinity stress in rice. *OsERF106MZ* is expressed in germinating seeds, primary roots, and developing flowers. Overexpression of *OsERF106MZ* led to retardation of growth, relatively high levels of both malondialdehyde (MDA) and reactive oxygen species (ROS), reduced catalase (CAT) activity, and overaccumulation of both sodium (Na^+^) and potassium (K^+^) ions in transgenic rice shoots. Additionally, the expression of *OsHKT1.3* was downregulated in the shoots of transgenic seedlings grown under both normal and NaCl-treated conditions, while the expression of *OsAKT1* was upregulated in the same tissues grown under NaCl-treated conditions. Further microarray and qPCR analyses indicated that the expression of several abiotic stress-responsive genes such as *OsABI5* and *OsSRO1c* was also altered in the shoots of transgenic rice grown under either normal or NaCl-treated conditions. The novel transcription factor OsERF106MZ negatively regulates shoot growth and salinity tolerance in rice through the disruption of ion homeostasis and modulation of stress-responsive gene expression.

**Supplementary Information:**

The online version contains supplementary material available at 10.1186/s12284-021-00525-5.

## Background

Soil salinity is one of the major constraints limiting crop growth and production. In general, soil with an electrical conductivity of saturated soil-paste extract (ECe) of 4 dS m^−1^ (equivalent to 40 mM NaCl) or more is considered to be saline (United State Department of Agriculture 2016). It is speculated that more than 20% of all cultivated land and almost half of all irrigated land face the problem of soil salinization (FAO, http://www.fao.org/home/en/). In plants, excessive salt uptake from the soil can lead to an imbalance of cellular ions, resulting in osmotic dehydration of the cells and ion toxicity. However, plants have evolved a sophisticated regulatory mechanism to deal with salinity stress. The osmotic signal can be transduced into the nucleus via both abscisic acid (ABA)-dependent (e.g., involving calcium-dependent protein kinases [CDPKs] and transcription factors [TFs] such as myelocytomatosis/myeloblastosis [MYC/MYB]) and ABA-independent (e.g., via C-repeat binding factor/dehydration-responsive element binding protein [CBF/DREB] TFs) pathways, which in turn activate the expression of downstream stress-responsive genes that confer tolerance to salinity stress in plants (for a review, see Kumar et al. 2013). Additionally, the regulation of sodium uptake, transport, and compartmentation is also important for plant survival under salinity stress. Several types of transporters, such as HKTs (high-affinity K^+^ transports), NHXs (Na^+^/H^+^ exchangers), and SOS (salt overly sensitive), have been shown to be involved in adjusting sodium homeostasis (Deinlein et al. 2014). When plants encounter salinity stress, these transporters act collectively to reduce the accumulation of cytosolic sodium, thereby alleviating sodium toxicity in cells (for a review, see Isayenkov and Maathuis 2019). Therefore, both TFs and the ion transporters function as key components that integrate signal transduction, stress-responsive gene expression, and sodium/potassium homeostasis to enhance salinity tolerance in plants.

TFs are proteins that regulate the transcription of target genes by directly binding to a specialized sequence known as a *cis*-regulatory (or *cis*-acting) element (CRE), in the promoter regions. Therefore, it can be expected that almost all TFs contain at least one DNA-binding domain (DBD) that recognizes and attaches to a specific CRE in the promoter region of target genes (Mitchell and Tjian 1989; Ptashne and Gann 1997). To date, at least 2296, 3308, 1891, and 2048 TFs have been found in *Arabidopsis* (*Arabidopsis thaliana*), maize (*Zea mays*), *indica* rice (*Oryza sativa* subsp. *indica*), and *japonica* rice (*Oryza sativa* subsp. *japonica*), respectively (Additional file 1: Figure S1). Notably, more than 45% of TFs in *Arabidopsis*, maize, and rice belong to the bHLH (basic helix-loop-helix), bZIP (basic leucine zipper), AP2/ERF (APETALA2/ethylene-responsive factor), C2H2 (Cys2/His2-type zinc finger protein), MYB, NAC (NAM, ATAF, and CUC), and WRKY families (Additional file 1: Figure S1), which implies that members of these seven families are extensively involved in the regulation of physiological processes in plants, including abiotic stress responses. For example, AtbHLH112 increases the expression of *POD* (*peroxidase*) and *SOD* (*superoxide dismutase*) genes to improve ROS scavenging ability, which confers tolerance to drought and salinity stresses in *Arabidopsis* (Liu et al. 2015). In rice, NAC022 plays a positive role in drought and salinity tolerance through the increased expression of two ABA biosynthetic genes (*OsNCED3* [*nine-cis-epoxycarotenoid dioxygenase 3*] and *OsPSY* [*phytoene synthase*]), signaling and regulatory genes (such as *OsDREB2a*), and late stress-responsive genes (such as *OsLEA3* [*late embryogenesis abundant 3*]) (Hong et al. 2016). Therefore, these previous studies illustrated that TF-regulated gene expression plays a pivotal role in the management of abiotic stresses among various plants.

AP2/ERFs comprise the second largest TF family in *Arabidopsis*, maize, and *japonica* rice and the largest TF family in *indica* rice. To date, at least 176, 261, 170, and 189 AP2/ERFs have been identified in *Arabidopsis*, maize, *indica* rice, and *japonica* rice, respectively (Additional file 1: Figure S1). AP2/ERFs are characterized by a conserved DBD known as the AP2/EREBP (APETALA2/ethylene-responsive element binding protein) domain, which consists of approximately 60 amino acid (aa) residues (Ohme-Takagi and Shinshi 1995). Based on the similarity and number of AP2/EREBP domains as well as the occurrence of additional domains, the AP2/ERFs can be broadly categorized into four major subfamilies: (1) the AP2 subfamily; (2) the RAV (related to ABA insensitive 3/viviparous 1) subfamily; (3) the CBF/DREB subfamily; and (4) the ERF subfamily, containing one AP2/EREBP domain with a conserved alanine residue at position 14 and a conserved aspartic acid at position 19 (Sakuma et al. 2002; Nakano et al. 2006). Based additionally on gene structure (e.g., exon–intron organization), the CBF/DREB subfamily can be further classified into 4 distinct groups (I-IV) in *Arabidopsis* and *japonica* rice, while the ERF subfamily can be further divided into 8 groups (V-X, VI-like, and Xb-like) in *Arabidopsis* and 11 groups (V-XIV and VI-like) in *japonica* rice (Nakano et al. 2006). In addition, CBF/DREB proteins regulate the transcription of target genes by binding to a CRE known as the CRT/DRE (C-repeat/dehydration-responsive element [5’-TACCGACAT-3’]) box in their promoters, but the ERF binds to the GCC (5’-AGCCGCC-3’) box (Ohme-Takagi and Shinshi 1995; Jiang et al. 1996). In *Arabidopsis*, several CBF/DREB-encoding genes have been extensively characterized and shown to be involved in abiotic stress responses. For example, CBF/DREB-IIIc-type TFs together with another cold-responsive TF, AtbHLH116/ICE1 (inducer of CBF expression 1), directly activate a majority of CRT/DRE-containing *CORs* (*cold responsive genes*), which contributes positively to cold tolerance (Chinnusamy et al. 2003; Zhao et al. 2016; Liu et al. 2018). Interestingly, AtERF006/RAP2.1 (related to AP2 1 [At6g46768]), a cold-induced CBF/DREB-IIa-type TF, acts as a repressor of CBF/DREB1-mediated cold acclimation, which negatively regulates cold tolerance (Dong and Liu 2010). These studies suggest that AP2/ERFs can antagonize each other to adjust plant growth in response to abiotic stresses.

Rice is one of the most important food crops in the world. However, rice is highly sensitive to salinity stress, especially at the young seedling stage. The threshold ECe for most cultivated rice varieties is 3 ds/m^−1^, and each 1% increase in ECe beyond this threshold can cause an approximately 12% decline in the yield of rice (Maas and Hoffman 1997). Therefore, efforts to decipher the regulatory mechanisms of salinity tolerance are essential for the development of salt-tolerant rice varieties. To identify which uncharacterized *OsERFs* may be involved in the salinity stress response, we analyzed the expression profiles of *OsERFs* from publicly available microarray datasets (GSE6901 and GSE14275), and several salinity stress-responsive *OsERFs* with an unknown function were found. Among these genes, the expression of *ethylene-responsive factor 106* (*OsERF106* [LOC_Os08g42550]) was upregulated under salinity stress conditions. *OsERF106* is a member of the rice ERF-Xc subgroup along with *OsERF105* (LOC_Os05g36100) and *OsERF107* (LOC_Os02g32140) (Nakano et al. 2006). The functions of rice ERF-Xc subgroup genes have not yet been characterized. Moreover, the annotation of putative *OsERF106* transcripts in the Rice Annotation Project Database (RAP-DB, http://rapdb.dna.affrc.go.jp/) are inconsistent with those in the Michigan State University Rice Genome Annotation Project Database (MSU RGAP, http://rice.plantbiology.msu.edu/). In this study, we isolated a cDNA encoding a previously unannotated OsERF106 and characterized its role in the rice plant response to salinity stress through a reverse genetic approach.

## Results

### Molecular Cloning and Structural Characterization of a Salinity Stress-Responsive TF Gene, OsERF106MZ

**Fig. 1:**
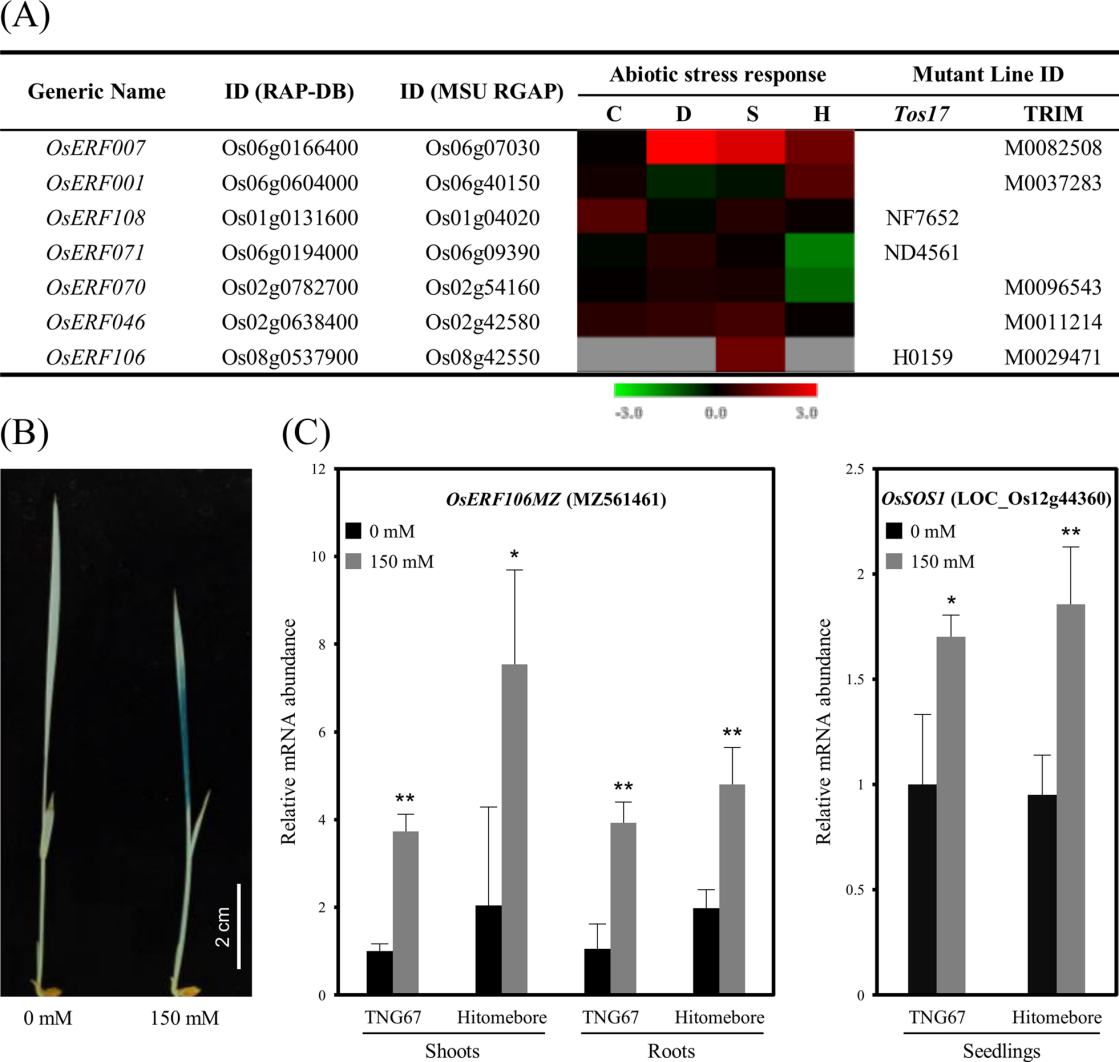
NaCl-enhanced *OsERF106MZ* expression. **a** Abiotic stress-responsive *OsERFs*. *Red* and *green* indicate increased and decreased gene expression, respectively. The scale bar shows log_2_-fold changes. C, cold stress; D, drought stress; S, salinity stress; H, heat stress. *Tos17*, Rice *Tos17* Insertion Mutant Database; TRIM, Taiwan Rice Insert Mutant Database. **b** Histochemical staining of the shoots in *OsERF106MZp::GUS* transgenic seedlings. **c** Quantification of *OsERF106MZ* and *OsSOS1* mRNA levels in Tainung 67 (TNG67) and Hitomebore seedlings by qPCR. The values are the mean ± SE of five biological replicates, each with two technical replicates. Asterisks indicate significant differences (*P < 0.05 and **P < 0.01) based on Student’s *t*-test. The seedlings used for the GUS staining (**b**) and qPCR (**c**) assays were grown on basal medium for 7 days and then transferred to basal medium containing 0 or 150 mM NaCl for an additional day

In silico analyses of microarray-based gene expression profiles indicated that the expression of *OsERF106* is upregulated under salinity stress but is undetectable under cold, drought, or heat stress (Fig. 1a). Notably, the annotation of putative OsERF106-encoding transcripts in the RAP-DB was different from that in the MSU RGAP. Two transcript variants of *OsERF106* (Os08t0537900-01 and Os08t0537900-02) were documented in the RAP-DB, while six alternative splice forms of *OsERF106* (LOC_Os08g42550.1 to LOC_Os08g42550.6) were described in the MSU RGAP (Additional file 1: Figure S2). Among these putative OsERF106-encoding transcripts, Os08t0537900-01 (as well as LOC_Os08g42550.2 to LOC_Os08g42550.6) encodes an O-fucosyltransferase (O-FucT) of 453 aa in length, but this enzyme does not contain an AP2/EREBP domain, a defining feature of the AP2/ERF proteins (Additional file 1: Figures S2 and S3). The coding sequence (CDS) of Os08t0537900-02 seems to be incorrect because it lacks an ATG-start codon and encodes an unknown protein without an AP2/EREBP domain (Additional file 1: Figures S2 and S4). LOC_Os08g42550.1 encodes a 541-aa protein that contains an O-FucT-like domain and an AP2/EREBP domain. However, the LOC_Os08g42550.1-encoded protein is homologous to AtO-FucT13 and GmO-FucT13 but is not homologous to any AtAP2/ERF or GmAP2/ERF (Additional file 1: Figures S2 and S5). This is unusual because no AP2/EREBP domain-containing O-FucT-like proteins have been identified to date. Furthermore, we were not able to amplify the full-length CDS of LOC_Os08g42550.1 by RT-PCR (reverse transcription-PCR). Therefore, we performed rapid amplification of cDNA ends-PCR (RACE-PCR) using gene-specific primers (GSPs) to clarify the CDS of *OsERF106*. The PCR-amplified product was approximately 1.3 kb in length, including a 5' untranslated region (5' UTR) of 351 bp, an open reading frame (ORF) of 648 bp encoding a previously unannotated OsERF106, and a 3' UTR of 333 bp. This previously unannotated *OsERF106* gene, designated *OsERF106MZ* (GenBank accession No. MZ561461), consisted of two exons, and the encoded protein contained a typical AP2/EREBP domain spanning residues 36 to 93 (Additional file 1: Figure S6). Phylogenetic analysis revealed that OsERF106MZ (together with OsERF105 [LOC_Os05g36100] and OsERF107 [LOC_Os02g32140]) is homologous to AtERF108/RAP2.6 (At1g43160) and AtERF113/RAP2.6L (At5g13330) but is not highly homologous to AtO-FucT13 or GmO-FucT13 (Additional file 1: Figure S7).

To verify whether the expression of *OsERF106MZ* was upregulated under salinity stress, histochemical staining was performed in *OsERF106MZp::β-glucuronidase* (*GUS*) transgenic rice. Under normal (0 mM NaCl) conditions, *GUS* was rarely expressed in the shoots of 8-day-old transgenic seedlings; however, its expression was obviously enhanced in response to 150 mM NaCl (Fig. 1b). Additionally, the expression pattern of *OsERF106MZ* was similar to that of *OsSOS1* (LOC_Os12G44360), a well-known salinity stress-responsive gene that was upregulated under 150 mM NaCl conditions in Tainung 67 (*Oryza sativa* L. *spp. japonica* cv. Tainung 67) and Hitomebore (*Oryza sativa* L. *spp. japonica* cv. Hitomebore) seedlings (Fig. 1c). These data indicate that *OsERF106MZ* is a salinity stress-responsive TF gene.

### Spatiotemporal OsERF106MZ Expression and OsERF106MZ Localization

**Fig. 2:**
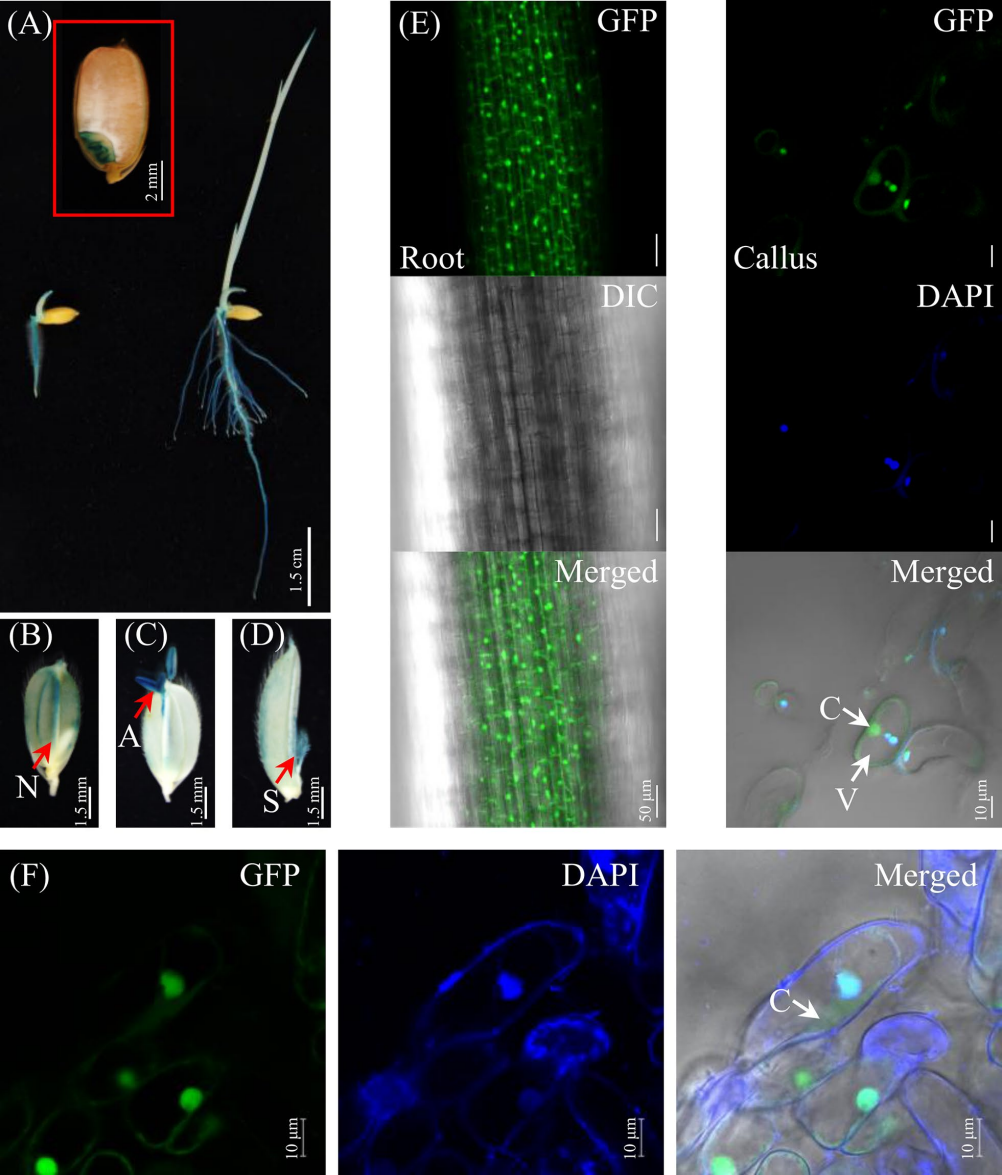
The spatiotemporal expression of *OsERF106MZ* and the subcellular localization of OsERF106MZ-GFP in stable rice transformants. **a** GUS staining of germinating seeds (insert) and post-germination-stage seedlings from an *OsERF106MZp::GUS* transformant. **b**–**d** GUS activity in developing flowers of an *OsERF106MZp::GUS* transformant. N, nerve; A, anther; S, stigma. **e** The presence of OsERF106MZ-GFP in the root vasculature (left panels) and callus cells (right panels). **f** Subcellular localization of OsERF106MZ-GFP in the NaCl-treated callus cells. The calli induced from the husk-removed seeds harboring *35Sp::OsERF106MZ-GFP* were treated with 150 mM NaCl for 24 h and then subjected to GFP visualization by confocal microscopy. C, cytosol; V, vacuole

The analysis of spatiotemporal gene expression trajectories and protein localization may offer valuable insights into gene function. Thus, we examined the spatiotemporal expression pattern of *OsERF106MZ* by detecting GUS signals in *OsERF106MZp::GUS* transgenic plants from the beginning of seed germination to the reproductive stage. After imbibition, the GUS signal was localized to the embryo of germinating *OsERF106MZp::GUS* transgenic seeds (Fig. 2a). After germination, *GUS* was predominantly expressed in the terrestrial tissues, such as the primary and seminal roots, of transgenic seedlings (Fig. 2a). Additionally, GUS signals were also observed in the lemma nerves, anthers, and stigmas during reproductive growth (Fig. 2b–d).

To examine the subcellular localization of OsERF106MZ, we transformed *OsERF106MZ-green fluorescent protein* (*GFP*) and *GFP-OsERF106MZ* constructs driven by the cauliflower mosaic virus (CaMV) 35S promoter into *Oncidium* ‘Sweet Sugar’ suspension cells using electroporation. Similar to the results obtained for GFP alone, both the OsERF106MZ-GFP and GFP-OsERF106MZ fusion proteins were ubiquitously distributed throughout the entire cell (Additional file 1: Figure S8). To further verify the subcellular localization of OsERF106MZ, a *35Sp::OsERF106MZ-GFP* construct was transformed into *japonica* rice cultivar Tainung 67 plants via stable *Agrobacterium*-mediated transformation. As shown in Fig. 2e, the OsERF106MZ-GFP fusion proteins were distributed throughout the cells of the root vasculature in transgenic rice seedlings. Indeed, the OsERF106MZ-GFP fusion protein not only colocalized with the nuclear 4′,6-diamidino-2-phenylindole (DAPI) stain but also was present in the cytosol of non-NaCl-treated callus cells or NaCl-treated callus cells (Fig. 2e, f). Therefore, these data reveal that OsERF106MZ, a salinity stress-responsive TF, does not specifically localize to the nucleus in rice cells.

### Overexpression of OsERF106MZ Reduces Plant Growth and Tolerance to Salinity Stress in Transgenic Rice Seedlings

**Fig. 3:**
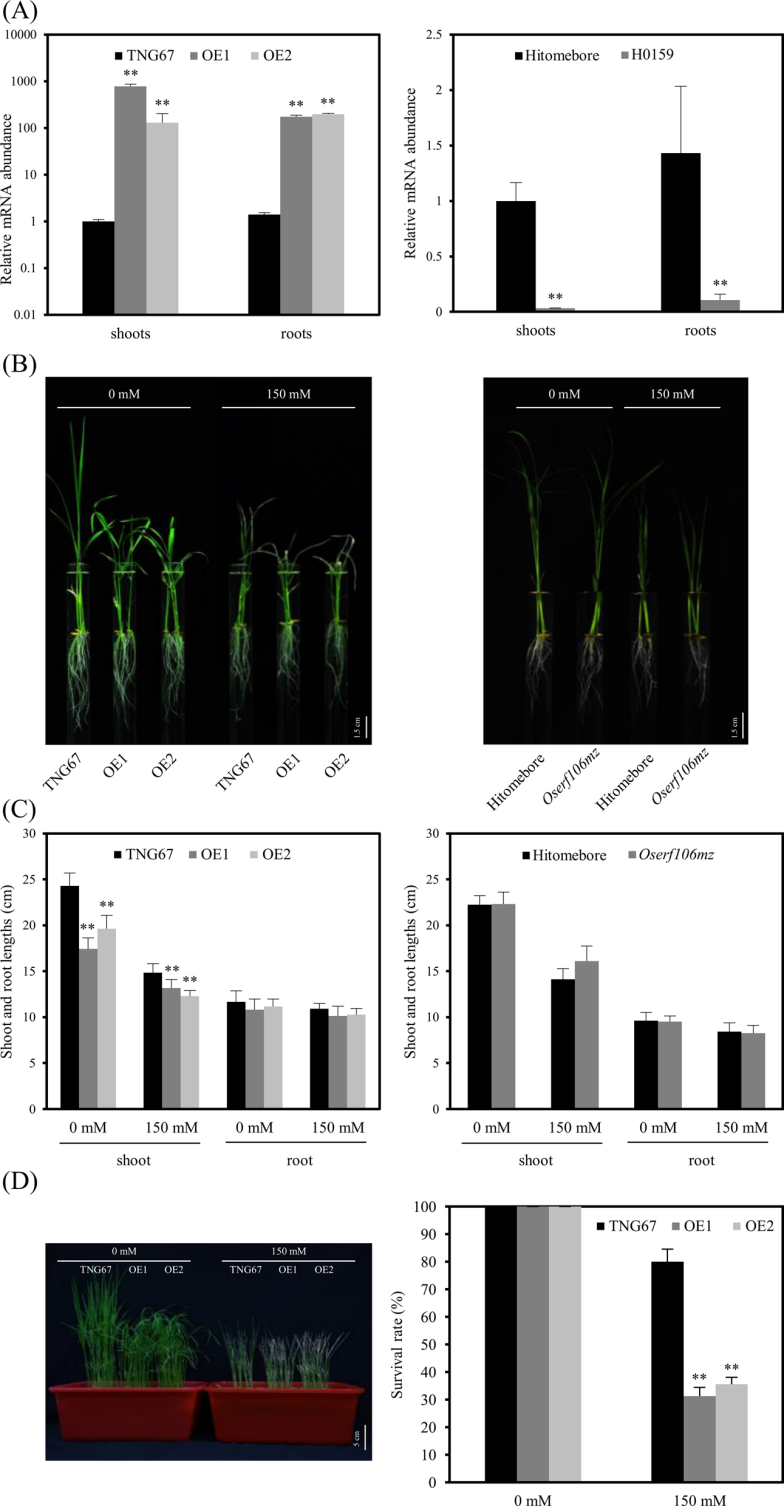
A comparison of growth patterns and salinity stress responses among *OsERF106MZ*-overexpressing transformants (OE1 and OE2), the *Oserf106mz* mutant, and the corresponding WT cultivars under normal (0 mM NaCl) and NaCl-treated (150 mM NaCl) conditions. **a** Quantification of *OsERF106MZ* mRNA levels in *OsERF106MZ*-overexpressing transformants, the *Oserf106mz* mutant, and the corresponding WT cultivars by qPCR. The positions of the primers are indicated by purple arrows in Additional file 1: Figure S9A. The primer sequences are listed in Additional file 2: Table S1. The seedlings were grown on basal medium for 11 days and then subjected to qPCR assays. **b** Morphological appearance of the *OsERF106MZ*-overexpressing transformants, *Oserf106mz* mutant, and the corresponding WT cultivars. **c** The shoot and root lengths of the *OsERF106MZ*-overexpressing transformants, *Oserf106mz* mutant, and the corresponding WT cultivars. **d** Morphological phenotype and survival rate of the *OsERF106MZ*-overexpressing transformants and its corresponding WT cultivars. The seedlings used for the assessment of morphological characteristics such as shoot and root lengths (**b** and **c**) and the calculation of survival rates (**d**) were grown on basal medium for 7 days and then transferred to basal medium containing 0 or 150 mM NaCl for an additional 4 and 6 days, respectively. The values of shoot and root lengths (**c**) are the mean ± SE of at least five biological replicates, each with two technical replicates. All survival rates (d) are the mean ± SE of 4 independent biological replicates. Each biological replicate contains at least 20 seedlings. Asterisks indicate significant differences (*P < 0.05 and **P < 0.01) in comparison to the corresponding WT based on Student’s *t*-test

To investigate the functional role of *OsERF106MZ* in regulating rice growth and development, a *35Sp::OsERF106MZ* construct was transformed into plants of the *japonica* rice cultivar Tainung 67, and homozygous *OsERF106MZ*-overexpressing transgenic rice plants were obtained in the T_2_ generation. Additionally, we searched the Rice *Tos17* Insertion Mutant Database and selected a retrotransposon insertion *Oserf106* mutant line, H0159. Based on its annotation, the retrotransposon is inserted into an intron of *OsERF106MZ*, which is equivalent to intron 7 of LOC_Os08g42550.1 (Additional file 1: Figure S9A). Homozygous H0159 lines were identified from the segregating population by genomic DNA genotyping PCR (Additional file 1: Figure S9B). Quantitative PCR (qPCR) analysis using the primer pair *OsERF106*-qF and *OsERF106*-qR, amplifying a 108 bp fragment downstream of the region encoding the AP2/EREBP domain, revealed that the mRNA level of *OsERF106MZ* was higher in transgenic rice plants harboring either *35Sp::OsERF106MZ* (OE1) or *35Sp::OsERF106MZ-GFP* (OE2) than in the corresponding wild-type (WT) Tainung 67 plants, while the mRNA level of *OsERF106MZ* was lower in the homozygous H0159 mutant than in the corresponding WT Hitomebore plant (Fig. 3a, Additional file 1: Figure S9A). Thus, the homozygous H0159 plant was used as an *Oserf106mz* mutant in this study. Incidentally, no significant difference in the mRNA levels of LOC_Os08g42550.s was observed between the *Oserf106mz* mutant and its corresponding WT Hitomebore plant when the universal primer pair *O-FucT*-qF and *O-FucT*-qR was employed to amplify a common fragment of 72 bp containing a sequence encoding part of O-FucT from the LOC_Os08g42550.s transcript (Additional file 1: Figure S9A and C).

Because the expression of *OsERF106MZ* is induced by salinity stress, we subsequently examined the differences in growth performance among the *OsERF106MZ*-overexpressing transformants (OE1 and OE2), the *Oserf106mz* mutant, and the corresponding WT cultivars under both normal and NaCl-treated conditions. As shown in Fig. 3b and c, the plant height of *OsERF106MZ*-overexpressing transgenic seedlings was significantly shorter than that of the corresponding WT Tainung 67 seedlings under either normal or NaCl-treated conditions, while the root length of *OsERF106MZ*-overexpressing transgenic seedlings was not different from that of WT Tainung 67 seedlings. In addition to the dwarf phenotype, *OsERF106MZ*-overexpressing transgenic seedlings also had a relatively low rate of survival under NaCl-treated conditions (Fig. 3d). Notably, no apparent difference in plant growth or morphology was observed between the *Oserf106mz* mutant and its corresponding WT Hitomebore seedlings under normal or NaCl-treated conditions (Fig. 3b and c).

**Fig. 4:**
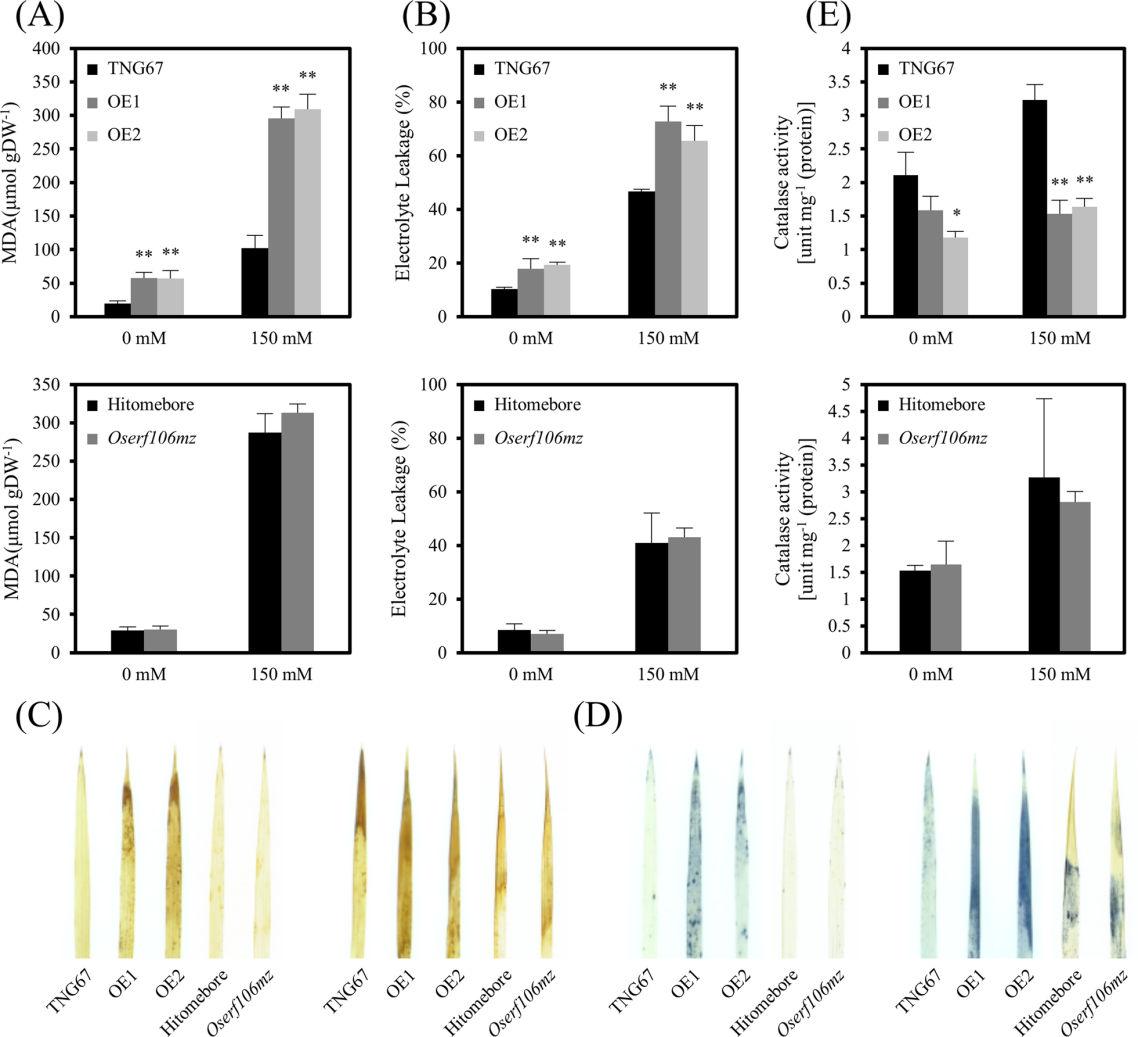
Analysis of MDA content, electrolyte leakage, ROS levels, and CAT activity in the shoots of the *OsERF106MZ*-overexpressing transformants, *Oserf106mz* mutant, and the corresponding WT cultivars under normal and NaCl-treated conditions. **a** MDA content. **b** Electrolyte leakage. **c** DAB staining. **d** NBT staining. **e** CAT activity. The values are the mean ± SE of five biological replicates, each with two technical replicates. Asterisks indicate significant differences (*P < 0.05 and **P < 0.01) in comparison to the corresponding WT based on Student’s *t*-test. For these assays, seedlings were grown on basal medium for 7 days and then transferred to basal medium containing 0 or 150 mM NaCl for an additional day

Phospholipids and membrane proteins are the major components of biological membranes in all living organisms. Under abiotic stress conditions, excessive lipid peroxidation can alter the composition or fluidity of membranes, further leading to membrane damage. Therefore, we examined the malondialdehyde (MDA, an index of lipid peroxidation) and relative electrolyte leakage (an index reflecting the changes of cell membrane permeability) levels among the *OsERF106MZ*-overexpressing transformants, the *Oserf106mz* mutant, and the corresponding WT cultivars. Under both normal and NaCl-treated conditions, the MDA content and electrolyte leakage in the shoots of *OsERF106MZ*-overexpressing transformants were significantly higher than those of the corresponding WT Tainung 67 plants (Fig. 4a and b). The relatively high level of MDA was also observed in the roots of *OsERF106MZ*-overexpressing transformants under normal conditions (Additional file 1: Figure S10A). Additionally, histochemical analyses with 3,3′-diaminobenzidine (DAB) and nitro blue tetrazolium (NBT) showed that the ROS levels in the leaves of *OsERF106MZ*-overexpressing transformants were higher than those in the WT Tainung 67 plants under either normal or NaCl-treated conditions (Fig. 4c and d). In contrast, CAT activity was significantly decreased in the shoots of *OsERF106MZ*-overexpressing transformants compared to that in the WT Tainung 67 plants under either normal or NaCl-treated conditions. Furthermore, NaCl-induced CAT activity was almost completely abolished in the shoots of *OsERF106MZ*-overexpressing transformants (Fig. 4e). Notably, no significant difference in ROS levels or CAT activity was observed between the *Oserf106mz* mutant and the corresponding WT Hitomebore seedlings under either normal or NaCl-treated conditions, which was similar to the morphological assessment results (Fig. 3 vs. Figure 4). Taken together, these data reveal that overexpression of *OsERF106MZ* in rice negatively regulates shoot growth and salinity stress tolerance, but its function in regulating these physiological processes may be redundant with those of other homologs.

### Overexpression of OsERF106MZ Leads to Excessive Accumulation of Na^+^ in Transgenic Rice Seedlings

**Fig. 5:**
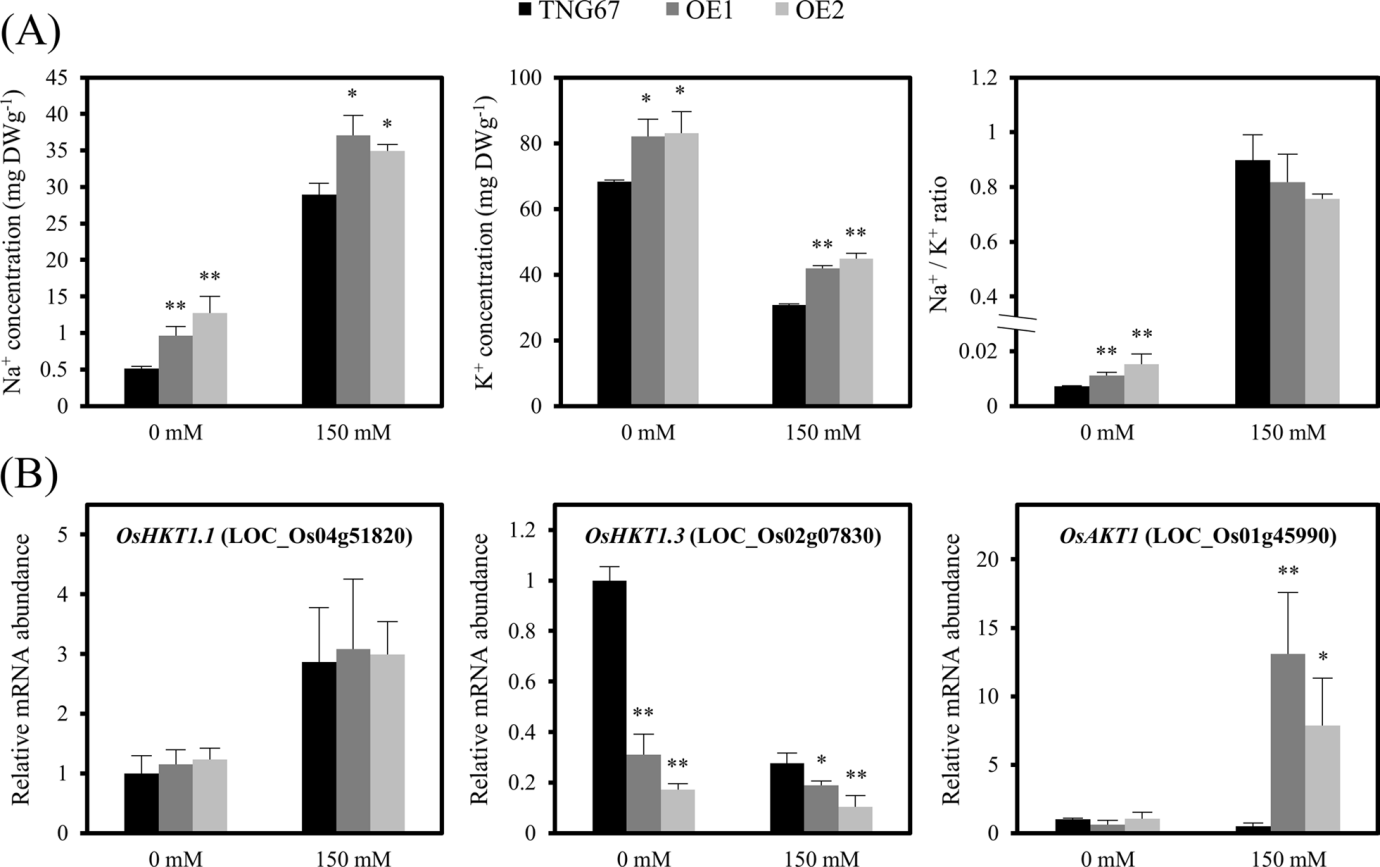
Overexpression of *OsERF106MZ* impairs the homeostasis of sodium and potassium in transgenic shoots. **a** Na^+^ and K^+^ contents and Na^+^/K^+^ ratio in the shoots of Tainung 67 and *OsERF106MZ*-overexpressing rice plants. **b** Quantification of *OsHKT1.1*, *OsHKT1.3*, and *OsAKT1* mRNAs in the shoots of Tainung 67- and *OsERF106MZ*-overexpressing rice plants. The values are the mean ± SE of at least four biological replicates, each with two technical replicates. Asterisks indicate significant differences (*P < 0.05 and **P < 0.01) in comparison to the corresponding WT (Tainung 67) based on Student’s *t*-test. The seedlings were grown on basal medium for 7 days and then transferred to basal medium containing 0 or 150 mM NaCl for an additional 4 days

To understand the nature of the relationship between plant growth and salinity stress responses in *OsERF106MZ*-overexpressing transgenic rice seedlings, we further examined the differences in Na^+^ and K^+^ levels between Tainung 67 and *OsERF106MZ*-overexpressing rice plants by using inductively coupled plasma-optical emission spectrometry (ICP-OES). Under normal conditions, Na^+^ levels were increased by at least 85% and K^+^ levels were increased by approximately 20% in both OE1 and OE2 shoots when normalized to the levels in the corresponding Tainung 67 shoots (Fig. 5a). These changes led to a higher Na^+^/K^+^ ratio in *OsERF106MZ*-overexpressing shoots grown under normal conditions. Similarly, the levels of Na^+^ and K^+^ were also higher in both OE1 and OE2 shoots than in the corresponding Tainung 67 shoots under NaCl-treated conditions. Moreover, the relatively high level of Na^+^ was also observed in the roots of OE plants under normal conditions (Additional file 1: Figure S10B). These data imply that OsERF106MZ may be involved in regulating sodium and potassium homeostasis. Accordingly, we examined the changes in the expression levels of several ion transport-related genes in *OsERF106MZ*-overexpressing shoots grown under normal or NaCl-treated conditions. As shown in Fig. 5b, no obvious differences in the expression of *OsHKT1.1* were found between the Tainung 67- and *OsERF106MZ*-overexpressing rice shoots under either normal or NaCl-treated conditions. However, the expression levels of *OsHKT1.3* and *OsAKT1* (a rice gene homologous to *Arabidopsis* K^+^ transporter 1) were significantly higher and lower, respectively, in *OsERF106MZ*-overexpressing rice shoots than in Tainung 67 shoots under normal and/or NaCl-treated conditions. Taken together, these results reveal that the overexpression of *OsERF106MZ* interferes with ion transport and subsequently causes the excessive accumulation of Na^+^ and K^+^ in the shoots, which may exacerbate ion toxicity, resulting in shoot growth retardation of the transgenic rice seedlings.

### Transcriptomic Analysis of OsERF106MZ-Overexpressing Transgenic Rice Shoots

**Fig. 6:**
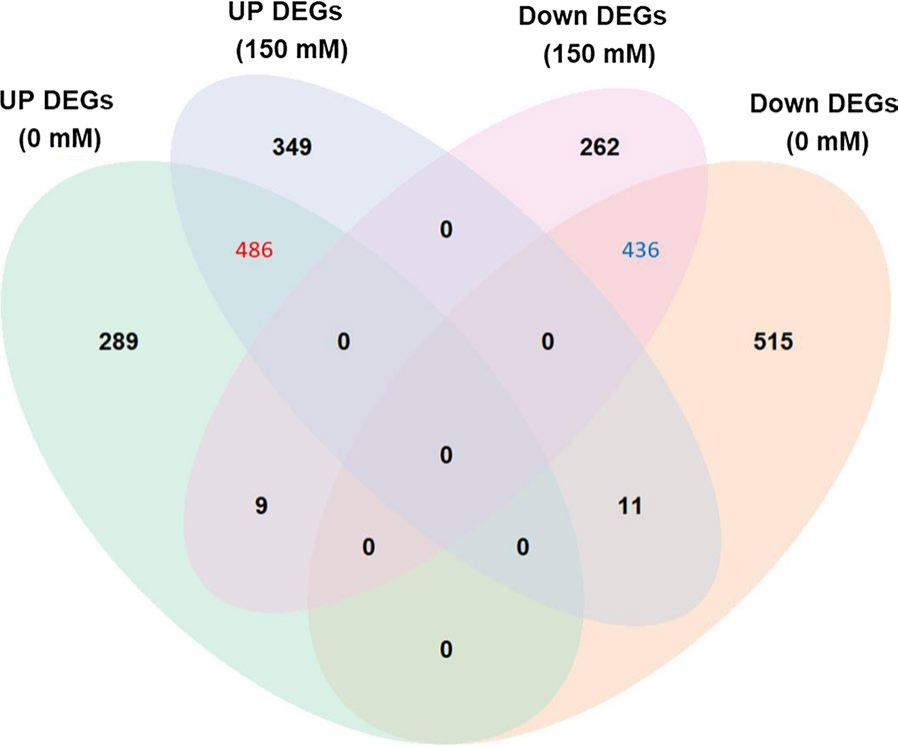
Venn diagram analysis of the DEGs between Tainung 67 and *OsERF106MZ*-overexpressing rice shoots

**Fig. 7:**
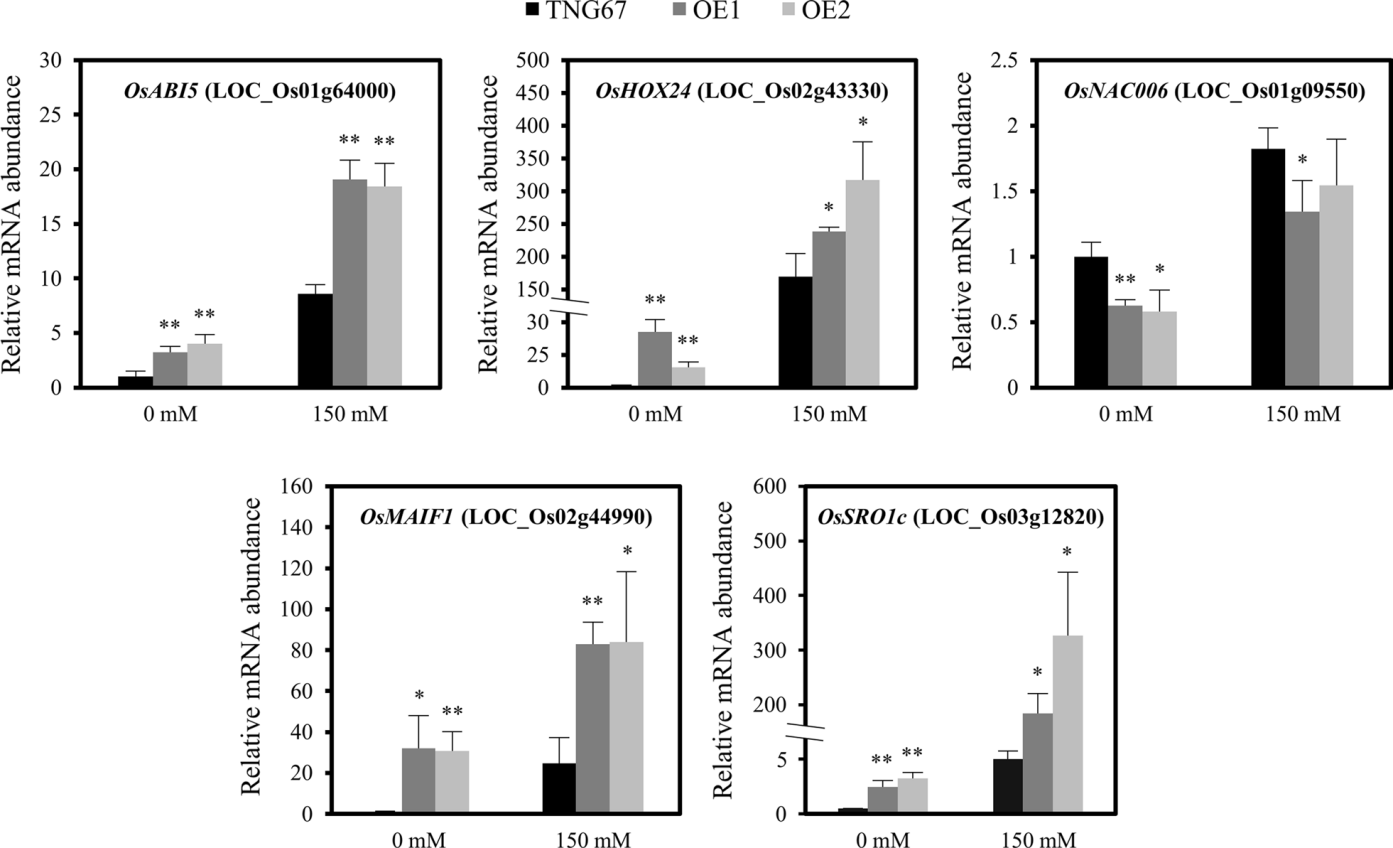
Quantification of abiotic stress-related DEG mRNAs in the shoots of two *OsERF106MZ*-overexpressing transgenic rice lines (OE1 and OE2) and Tainung 67 by qPCR. The values are the mean ± SE of five biological replicates, each with two technical replicates. Asterisks indicate significant differences (*P < 0.05 and **P < 0.01) in comparison to the corresponding WT (Tainung 67) based on Student’s *t*-test. The total RNA used in the qPCR and microarray assays was isolated from the same shoots of 7-day-old seedlings treated with 0 or 150 mM NaCl for 1 day

To gain better insight into the molecular mechanisms underlying *OsERF106MZ*-mediated responses to salinity stress in rice shoots, we performed a comparative transcriptomic analysis of Tainung 67- and *OsERF106MZ*-overexpressing shoots under both normal and NaCl-treated conditions. After background correction and normalization, a total of 1746 and 1553 differentially expressed genes (DEGs) were identified from the shoots of *OsERF106MZ*-overexpressing transgenic rice seedlings grown under normal and NaCl-treated conditions, respectively (Fig. 6, Additional file 3: Data S1). Among these genes, 784 and 846 DEGs were upregulated in the shoots of *OsERF106MZ*-overexpressing transgenic rice seedlings grown under normal and NaCl-treated conditions, respectively, while 962 and 707 DEGs were downregulated (Fig. 6, Additional file 3: Data S1). Additionally, a four-way Venn diagram showed that a total of 922 DEGs were common to the shoots of *OsERF106MZ*-overexpressing transgenic rice seedlings grown under both conditions, among which 486 and 436 DEGs were consistently up- and downregulated, respectively, under both conditions (Fig. 6, Additional file 4: Data S2). As expected, several abiotic stress-related genes such as *OsABI5* (*ABA-insensitive 5*), *OsMAIF1* (an F-box protein gene), and *OsSRO1c* (*similar to RCD* [*radical-induced cell death*] *One 1c*), were found among these commonly regulated DEGs. Accordingly, a qPCR assay was conducted to verify the microarray data. As shown in Fig. 7, the mRNA levels of *OsABI5*, *OsHOX24* (rice homeodomain-leucine zipper I subfamily member), *OsMAIF1*, and *OsSRO1c* were higher in *OsERF106MZ*-overexpressing shoots than in Tainung 67 shoots under both conditions, while the mRNA level of *OsNAC006* was lower in *OsERF106MZ*-overexpressing shoots than in Tainung 67 shoots. The results obtained by qPCR were consistent with the microarray data. In addition, gene ontology analysis revealed that the major ontological categories of the commonly regulated DEGs were ‘biological regulation’ and ‘cell wall organization or biogenesis’ under the ‘biological process’ category and ‘electron carrier activity’ and ‘transcription regulator activity’ under the ‘molecular function’ category (Additional file 1: Figure S11). Incidentally, further promoter analysis indicated that 50 and 23 common up- and downregulated DEGs, respectively, contained at least one GCC box, an ERF-binding CRE, in their 1-kb promoter region, including *OsMAIF1* (Additional file 4: Data S2).

## Discussion

### Accurate Annotation of OsERF106

Accurate annotation of gene structure is an initial step toward functional analysis of its roles in regulating plant growth, development, and responses to stress. Sometimes, it is not easy to accurately predict the coding region of a gene owing to the sequencing coverage and quality or an overlap of two individual genes in their coding regions. The AP2/ERF proteins comprise one of the largest TF families in plants. The AP2/ERF family is defined by containing one or two AP2/EREBP homology domains, which are responsible for sequence-specific DNA binding (Ohme-Takagi and Shinshi 1995; Nakano et al. 2006). As noted by Nakano et al. (2006), the members of the ERF subfamily can be further subdivided into 8 (V-X, VI-like, and Xb-like) and 11 groups (V-XIV and VI-like) in *Arabidopsis* and *japonica* rice, respectively, according to their gene structure and conserved motifs (Nakano et al. 2006). Most of these groups are present in both *Arabidopsis* and *japonica* rice, indicating that the structural evolution and functional diversification of the plant ERF subfamily may predate monocot/dicot divergence. Additionally, it has been reported that most of the *AtERF* genes possess no introns, and only a few of these genes contain a single intron (Sakuma et al. 2002; Nakano et al. 2006). These findings imply that the structure and function of the *OsERF* gene should be similar to those of its homologs in *Arabidopsis* and rice. In the present study, several abiotic stress-responsive *OsERF* genes of unknown function were found by analyzing publicly available microarray datasets (GSE6901 and GSE14275). Among these genes, the expression of the *OsERF106* gene was upregulated under salinity stress (Fig. 1a). However, the *OsERF106* gene was differently annotated in the RAP-DB and MSU RGAP. Two (Locus tag: Os08t0537900-01 and Os08t0537900-02) and six (Locus tag: LOC_Os08g42550.1 to LOC_Os08g42550.6) putative *OsERF106* transcript variants in the RAP-DB and MSU RGAP, respectively, were recorded (Additional file 1: Figure S2). Among these putative *OsERF106* loci, Os08t0537900-01 as well as LOC_Os08g42550.2 to LOC_Os08g42550.6 contain six exons and five introns, while LOC_Os08g42550.1 consists of eight exons and seven introns. Notably, it has been reported that *OsERF106* is highly homologous to *OsERF105* (LOC_Os05g36100) and *OsERF107* (LOC_Os02g32140), which belong to the Xc subgroup of the rice ERF subfamily (Nakano et al. 2006). Additionally, as indicated on the MSU RGAP website, the *OsERF105* (LOC_Os05g36100) and *OsERF107* (LOC_Os02g32140) genes are orthologous to *AtERF108/RAP2.6* (At1g43160) and *AtERF113/RAP2.6L* (At5g13330), two members of the X group within the *Arabidopsis* ERF subfamily. However, almost all of the *ERF* genes in the *Arabidopsis* X group as well as the *OsERF105* (LOC_Os05g36100) and *OsERF107* (LOC_Os02g32140) genes are composed of two exons and one intron. Therefore, the gene structure of the Os08t0537900-01 and LOC_Os08g42550.s loci does not seem to conform to the conventional rules of *ERF* genes possessing one intron at most and sharing high structural similarity with their homologs. Although the exon–intron organization of the Os08t0537900-02 locus conforms to these rules, its CDS is not consistent with the known characteristics of eukaryotic genes because it lacks an ATG start codon (Additional file 1: Figures S2 and S4).

Beyond their gene structure or the presence of an intact CDS, the proteins encoded by these putative *OsERF106* loci are not easy to interpret. Among these putative *OsERF106* loci, Os08t0537900-01 and LOC_Os08g42550.2 to LOC_Os08g42550.6 encode an O-fucosyltransferase (O-FucT) that does not contain an AP2/EREBP domain, which is a defining feature of AP2/ERF proteins (Additional file 1: Figures S2 and S3). The CDS of Os08t0537900-02 encodes an unknown protein that also lacks the AP2/EREBP domain, even though its CDS lacks an ATG-start codon (Additional file 1: Figures S2 and S4). LOC_Os08g42550.1 encodes an AP2/EREBP domain-containing O-FucT-like protein; however, this protein is homologous to AtO-FucT13 and GmO-FucT13 and is not homologous to any AtAP2/ERFs or GmAP2/ERFs, similar to the situation in the proteins encoded by Os08t0537900-01 and other LOC_Os08g42550 loci (Additional file 1: Figures S2, S3, and S5). Notably, O-FucT is an enzymatic protein and is not a TF, and it is responsible for transferring O-fucose from a donor substrate, such as guanosine diphosphate-fucose (GDP-fucose), to a protein (Ma et al. 2006; Tu et al. 2013). Therefore, a DBD, such as an AP2/EREBP domain, does not seem to be required for the enzymatic function of the O-FucT protein. In addition, qPCR analysis revealed that the RNA levels of LOC_Os08g42550 in *Oserf106mz*, a *Tos17* mutant H0159 line harboring a *Tos17* insertion within intron 7 of the LOC_Os08g42550.1 locus, were not significantly different from those in WT Hitomebore plants when the primer pair *O-FucT*-qF and *O-FucT*-qR was used to amplify a common fragment of 72 bp containing a sequence encoding part of O-FucT from LOC_Os08g42550.s (Additional file 1: Figure S9A and C). Based on these unusual annotation results, we speculated that O-FucT is encoded by an individual gene upstream of the *OsERF106* gene and is not encoded by part of the *OsERF106* gene. Accordingly, RACE-PCR using GSPs was performed to clarify the CDS of the *OsERF106* gene. The RACE PCR-amplified *OsERF106*, designated *OsERF106MZ*, comprised two exons separated by an intron of 753 bp, and the encoded protein included an AP2/EREBP domain with conserved alanine and aspartic acid residues (Additional file 1: Figure S6). Additionally, OsERF106MZ is homologous to OsERF105 (LOC_Os05g36100), OsERF107 (LOC_Os02g32140), and their *Arabidopsis* homologs AtERF108/RAP2.6 (At1g43160) and AtERF113/RAP2.6L (At5g13330) but is not highly homologous to AtO-FucT13 and GmO-FucT13 (Additional file 1: Figure S7). In fact, the CDS of *OsERF106MZ* is almost identical to the CDS of Os08t0537900-02, except for (1) an 11 bp insertion at the beginning of exon 2 in the *OsERF106MZ* gene, (2) a 31 bp insertion at the end of exon 2 in the *OsERF106MZ* gene, and (3) a 3' UTR downstream of the TAA stop codon of the *OsERF106MZ* gene (Additional file 1: Figure S12). Notably, OsERF106MZ protein does not specifically localize to the nucleus, presumably due to the lack of a nuclear localization signal (NLS); likewise, both the OsERF105 (LOC_Os05g36100) and OsERF107 (LOC_Os02g32140) proteins lack an NLS (Additional file 1: Figure S13).

### OsERF106MZ, a Member of the Rice ERF-X Group, Negatively Regulates the Salinity Stress Response by Disrupting ion Homeostasis and Modulating Stress-Responsive Gene Expression

The AP2/ERF TF family plays an important role in regulating many aspects of plant growth and development (Licausi et al. 2013). Among the four AP2/ERF subfamilies, the role of CBF/DREB proteins in abiotic stresses has been extensively examined because several members of this subfamily, such as CBFs/DREB1s and DREB2s, play a major role in cold, drought, or salinity tolerance through the direct regulation of stress-responsive genes, including *COR15a* and *COR78/RD29A* (Lata and Prasad 2011; Mizoi et al. 2012; Xie et al. 2019). In addition to these members of the CBF/DREB subfamily, a growing number of members within the ERF subfamily, especially in the ERF-X group, have emerged as crucial regulators of various stress responses. In *Arabidopsis* and rice, there are 8 (*AtERF108* to *AtERF115*) and 13 (*OsERF98* to *OsERF107*, *OsERF118*, *OsERF124*, and *OsERF125*) genes belonging to the ERF-X group, respectively (Nakano et al. 2006). In *Arabidopsis*, overexpression of *AtERF108/RAP2.6* (At1g43160) causes hypersensitivity to ABA and abiotic stresses during seed germination and early seedling development (Zhu et al. 2010). AtERF111/ABR1 (ABA repressor 1 [At5g64750]) acts as a transcriptional activator to regulate the wounding response (Bäumler et al. 2019). *AtERF113/RAP2.6L* (At5g13330) overexpression delays waterlogging-induced premature senescence, probably through an *ABI1*-mediated ABA signaling pathway (Liu et al. 2012a, b). *Arabidopsis* seedlings carrying a mutation in *AtERF115* (At5g07310) are more sensitive to 75 mM and 100 mM NaCl than WT seedlings (Krishnamurthy et al. 2017). Therefore, these studies have illustrated or implied the importance of plant ERF-X group members in regulating abiotic stress responses. Notably, more than half of the rice ERF-X group members are also abiotic stress-responsive genes. Among the 13 members of the rice ERF-X group, 9 genes are upregulated under different abiotic stress conditions, including *OsERF106*, which is specifically upregulated by salinity stress (for detail, see Mishra et al. 2013). In addition, almost all of these abiotic stress-responsive OsERF-X group genes are commonly expressed during both seed and reproductive developmental stages (Mishra et al. 2013). Although the expression profile of rice ERF-X group genes in response to abiotic stresses has been investigated in detail, their functional roles in regulating abiotic stress responses are still poorly understood. In this article, we examined the roles of *OsERF106MZ*, a salinity stress-responsive OsERF-Xc-type gene, in regulating the growth and salinity stress response of rice plants. As shown in Figs. 1 and 2a–d, histochemical GUS staining and qPCR assays confirmed that the expression of *OsERF106MZ* is enhanced by NaCl and is detectable in germinating seeds and developing flowers (a reproductive tissue), similar to the findings of Mishra et al. (2013). The overexpression of *OsERF106MZ* in rice can cause a severe retardation of shoot growth accompanied by relatively high levels of both MDA and ROS and a depression of CAT activity; however, no obvious differences in these morphological, physiological, and biochemical traits were found between the shoots of the *Oserf106mz* mutant and its corresponding WT Hitomebore seedlings (Figs. 3 and 4). Further analysis revealed that *OsERF106MZ*-overexpressing transgenic rice seedlings accumulated excess Na^+^ and K^+^ ions in whole plants and shoots, respectively (Fig. 5a, Additional file 1: Figure S10B). Moreover, the expression of *OsHKT1.3* was downregulated in *OsERF106MZ*-overexpressing shoots under both normal and NaCl-treated conditions, while the expression of *OsAKT1* was upregulated in transgenic shoots under NaCl-treated conditions (Fig. 5b). The *OsHKT1.s*-encoded proteins are Na^+^-selective transporters, which are mostly located at the plasma membrane in xylem parenchyma cells (Hamamoto et al. 2015). OsHKT1.s proteins play a critical role in retrieving Na^+^ from xylem sap, which prevents Na^+^ overaccumulation in shoots. In addition, *OsAKT1* encodes an inward K^+^ channel that specifically localizes at the plasma membrane (Fuchs et al. 2005; Li et al. 2014). The expression of *OsAKT1* is downregulated in both the shoots and roots of rice seedlings under salinity stress (Fuchs et al. 2005). However, the overexpression of *OsAKT1* significantly increases the content of K^+^ in these tissues under osmotic/drought stress (Ahmad et al. 2016). Therefore, we speculated that the overaccumulation of Na^+^ and K^+^ ions in *OsERF106MZ*-overexpressing shoots may partially result from the interference of *OsERF106MZ* in the expression of *OsHKT1.3* and *OsAKT1*, which increases the toxicity of ions to cells and, thus, retards shoot growth in transgenic rice. In fact, a number of TFs have already been demonstrated to be involved in coordinately managing ion homeostasis and salinity stress responses. For example, OsbHLH035 mediates the expression of *OsHKT1.3* and *OsHKT1.5* in the aerial and terrestrial tissues of rice seedlings, respectively, which fine-tunes the response to salinity stress (Chen et al. 2018). OsSTAP1, a rice ERF-VIIa subgroup member, increases the expression of *OsHKT8* and accordingly reduces the content of Na^+^ ions in the shoots of rice plants, which contributes to the ability of rice plants to tolerate salinity stress (Wang et al. 2020). Thus, the function of OsERF106MZ seems to also be involved in the TF-mediated regulation of ion homeostasis in rice.

In addition to the regulation of ion homeostasis, the expression of several stress-responsive genes was altered in *OsERF106MZ*-overexpressing shoots under either normal or NaCl-treated conditions. Among these genes, the expression of *OsABI5*, *OsHOX24*, *OsMAIF1*, and *OsSRO1c* was consistently upregulated, while the expression of *OsNAC006* was consistently downregulated in *OsERF106MZ*-overexpressing shoots under both conditions (Fig. 7). *OsABI5* encodes a nuclear-localized bZIP-type TF whose expression is induced by ABA and high salinity but downregulated by drought and cold (Zou et al. 2008). The overexpression of *OsABI5* leads to an increase in sensitivity to salinity stress, while the repression of *OsABI5* expression improves stress tolerance in transgenic rice. Additionally, rice transgenic lines overexpressing *OsHOX24* exhibit extreme susceptibility to abiotic stresses at the seed germination stage and show severe retardation of seedling growth under salinity and desiccation stresses (Bhattacharjee et al. 2017). *OsSRO1c*, a direct target gene of SNAC1 (stress-responsive NAC 1), is involved in the oxidative stress response (You et al. 2013). The activity of enzymatic antioxidants, such as CAT, is significantly suppressed in *OsSRO1c*-overexpressing transgenic rice, and this transgenic rice is hypersensitive to oxidative stress. *OsMAIF1* is a rice F-box domain gene, and its expression is rapidly induced by abiotic stresses (Yan et al. 2011). The overexpression of *OsMAIF1* negatively regulates the abiotic stress tolerance but positively regulates the root growth of transgenic rice. Thus, we speculated that OsERF106MZ acts as a negative regulator of salinity stress via the disruption of ion homeostasis and the modulation of stress-responsive gene expression. Incidentally, despite excessive NA^+^ accumulation, *OsERF106MZ*-overexpressing transgenic seedlings do not show significant growth retardation of the roots. We suspected that the adverse effects of Na^+^ excess on the root growth of transgenic rice overexpressing *OsERF106MZ* may be masked by the *OsMAIF1*-mediated promotion of root elongation.

### The OsERF106MZ-Mediated Negative Regulation of Salinity Stress Responses may Provide a Balance That Adjust Plant Responses to Salinity Stress

In *Arabidopsis*, several CBF/DREB-IIIc-type TFs act as positive regulators of cold tolerance (for a review, see Xie et al. 2019). Notably, several CBF/DREB-IIa-type TFs, such as AtERF011/DEAR1 (DREB and EAR motif protein 1 [At3g50260]) and AtERF006/RAP2.1 (At1g46768), are also upregulated during cold acclimation and serve as negative regulators of the cold stress response (Fowler and Thomashow 2002). AtERF011/DEAR1 and AtERF006/RAP2.1 likely act upstream and downstream, respectively, of CBF/DREB-IIIc-type TFs (Tsutsui et al. 2009; Dong and Liu 2010). Overexpression of *AtERF011/DEAR1* suppressed the cold-induced expression of *CBFs/DREBs* and resulted in drastically reduced freezing tolerance (Tsutsui et al. 2009). Similarly, *Arabidopsis* plants overexpressing *AtERF006/RAP2.1* exhibited enhanced sensitivity to cold stress (Dong and Liu 2010). In fact, the TF-mediated negative regulation of abiotic stress responses is also found ubiquitously in crops. In *Brassica napus*, the early-expressed Group I DREBs switch on the CRT/DRE-mediated signaling pathway to increase plant tolerance to cold stress, whereas the late-expressed Group II DREBs competitively inhibited Group I DREB function to maintain a balance between plant growth and the cold stress response (Zhao et al. 2006). In the present study, rice seedlings overexpressing *OsERF106MZ*, a salinity-induced ERF-Xc-type TF, exhibited decreased tolerance to salinity stress with an increased Na^+^/K^+^ ratio in the shoots, which was quite similar to the findings in rice plants overexpressing *OsERF922* (LOC_Os01g54890), a salinity-induced ERF-IXa-type TF (Liu et al. 2012a, b). Therefore, the negative regulation of abiotic stress responses by abiotic stress-induced TF repressors in plants seems to provide a balance that minimizes the adverse effects of prolonged stress responses (Xie et al. 2019).

## Conclusions

In the present study, we isolated a novel *OsERF106*, designated *OsERF106MZ*, by using RACE-PCR. The expression of *OsERF106MZ* is induced by salinity stress. The overexpression of *OsERF106MZ* leads to an overaccumulation of Na^+^ and K^+^ ions in the shoots of transgenic rice, resulting in a severe retardation of shoot growth. Moreover, the overexpression of *OsERF106MZ* interferes with the expression of *OsHKT1.3*, *OsAKT1*, and several stress-responsive genes under normal or NaCl-treated conditions. All of the results suggest that *OsERF106MZ* negatively regulates salinity tolerance by disrupting ion homeostasis and modulating stress-responsive gene expression.

## Methods

### Plant Materials, Growth Conditions, and Experimental Methods

The *Oserf106mz* mutant (*Tos17* line H0159) and its corresponding WT cultivar (*Oryza sativa* L. *spp. japonica* cv. Hitomebore) were obtained from the National Institute of Agrobiological Sciences (NIAS, Japan). Two independent *OsERF106MZ*-overexpressing transgenic rice lines (*35Sp::OsERF106MZ* [OE1] and *35Sp::OsERF106MZ-GFP* [OE2]) were generated by *Agrobacterium*-mediated transformation. In all experiments, the seeds were treated with 1.5% (v/v) commercial bleach for 30 min, rinsed twice with sterile water for 30 min each time, and subsequently subjected to imbibition at 37 °C for 3 days in the dark. After imbibition, the germinating seeds were grown on a wire stand in a beaker at 28/24 °C (day/night) under long-day (16-h light/8-h dark) conditions with a light intensity of approximately 100 µE s^−1^ m^2^. The basal medium used in all of the experiments was half-strength Kimura B solution (Yoshida et al. 1976).

### Isolation of Rice OsERF106MZ Gene

The full-length cDNA of *OsERF106MZ* gene was obtained from 5' and 3' cDNAs generated using a rapid amplification of cDNA ends (RACE) system (Clontech). Phylogenetic analysis revealed that OsERF106 (LOC_Os08g42550) was highly homologous to OsERF105 (LOC_Os05g36100) and OsERF107 (LOC_Os02g32140) based on the sequence similarity of the AP2/EREBP domains (Nakano et al. 2006). Therefore, the gene-specific primers (GSPs) used in the 5' and 3' RACE experiments were located downstream of the region encoding the AP2/EREBP domain of *OsERF106* (LOC_Os08g42550.1), the nucleotide sequence of which was highly divergent from those of *OsERF105* and *OsERF107* (Additional file 1: Figure S14). The primer positions and sequences are shown in Additional file 1: Figure S14 and Additional file 2: Table S1, respectively.

### RNA Extraction, cDNA Synthesis, and Quantitative PCR (qPCR)

Total RNA was extracted from various tissues of rice using an RNeasy Plant Mini Kit (Qiagen) according to the manufacturer’s instructions. To minimize genomic DNA contamination, up to 8 µg of total RNA was treated after extraction with Turbo DNA-*free*™ DNase (Ambion) following the manufacturer’s instructions, and 3 µg of DNase-treated total RNA was then subjected to cDNA synthesis using the SuperScript™ III first-strand synthesis system (Invitrogen) according to the manufacturer’s instructions. qPCR was carried out in an ABI 7500 system using the SYBR® Green PCR Master Mix Kit (Applied Biosystems [ABI]). The initial amount of template cDNA in each amplification reaction was 10 µg. At least three independent biological replicates were performed for each experiment, and *OsACTIN1* (LOC_Os03g50885) was used as an internal control for qPCR normalization. The 2^−ΔΔCT^ method was used to transform threshold cycle values (Ct) into normalized relative abundance values of mRNA. The sequences of primers used for qPCR are presented in Additional file 2: Table S1.

### Transgene Constructs and Isolation of Transgenic Rice

The full-length CDSs of *OsERF106MZ* and *GFP* were PCR-amplified with or without stop codons and cloned into the pGEM-T Easy vector (Promega). As shown in parentheses below, these fragments were subcloned into the binary pCAMBIA-1300 vector, in which their expression was driven by a CaMV 35S promoter (*35Sp::OsERF106MZ* [OE1] and *35Sp::OsERF106MZ-GFP* [OE2]). After the constructs were confirmed by sequencing, they were transformed into the rice cv. Tainung 67 background for subcellular OsERF106MZ localization and functional assays. Because the stable T_0_ rice transformants were chimeric and to avoid the undesirable side effects of multiple T-DNA insertions, T_1_ seeds were harvested from individual panicles of independent transformant lines that showed a 3:1 ratio of hygromycin resistance and sensitivity normalized to seed viability and were used to further screen for homozygous transgenic rice. Additionally, the 2.3-kb *OsERF106MZ* promoter was cloned into pCAMBIA-1305.1 (*OsERF106MZp::GUS*), and the construct was transformed into the rice cv. Tainung 67 background for the investigation of the spatiotemporal pattern of *OsERF106MZ* expression. The spatiotemporal *OsERF106MZ* expression and subcellular OsERF106MZ localization assays were carried out as previously described (Chen et al. 2017).

### Measurement of MDA Content and Electrolyte Leakage

For the MDA and electrolyte leakage assays, the seedlings were transferred to basal medium supplemented with 0 or 150 mM NaCl after growing for 7 days in beakers. Twenty-four hours later, the seedlings were used as initial materials for the measurement of MDA content or electrolyte leakage. The MDA content was determined following the method of Esterbauer and Cheeseman (1990). The aerial parts of the plant material were excised and vacuum dried overnight. Up to 0.1 g of the dried samples was added to 0.4 mL of 5% (w/v) trichloroacetic acid (TCA). After homogenization, the mixture was centrifuged at 10,000×*g* for 5 min at 20 °C. Then, 0.1 mL of the supernatant was added to 0.4 mL of 0.5% (w/v) thiobarbituric acid (TBA) dissolved in 20% (w/v) TCA, followed by incubation for 30 min at 95 °C in a water bath. After incubation, the mixture was removed from the water bath and centrifuged at 3000×*g* for 10 min at 4 °C. The absorbance of the supernatant at 450, 532, and 600 nm was measured with a spectrometer. The MDA content was calculated as follows: MDA (µmol g^−1^ dry weight [DW]) = [6.452 × (A532 − A600) − 0.56 × A450] × 20 ÷ DW (g). Electrolyte leakage was measured according to the method described by Bajji et al. (2002). The aerial parts of the plant material were washed twice with distilled water and then cut into 0.25- to 0.5-cm-long pieces. An approximately 0.05-g sample of the leaf pieces was placed in a boiling tube filled with 50 mL of distilled water, which was then incubated in the dark for 24 h to allow the diffusion of electrolytes. Following incubation for 24 h, the initial electrical conductivity (R1) in the solution was determined using a conductivity meter (model 3173R, JENCO, China). Thereafter, all leaf pieces were subjected to autoclaving at 121 °C under a pressure of 15 pounds per square inch for 20 min to release the electrolytes completely, and the electrical conductivity (R2) in the solution was measured after 1 h of equilibration at room temperature. The percentage of electrolyte leakage was calculated as follows: (R1/R2) × 100%.

### 3,3′-Diaminobenzidine (DAB) and Nitro Blue Tetrazolium (NBT) Staining Assays

For the DAB and NBT staining assays, the seedlings were transferred to basal medium supplemented with 0 or 150 mM NaCl after growing for 7 days in beakers. Twenty-four hours later, the 2nd leaf of each plant was excised and subjected to DAB staining or NBT staining. For DAB and NBT staining, leaves were incubated in a DAB (0.1% [w/v] DAB, 10 mM Na_2_HPO_4_, 0.05% Tween 20, pH 3.8) or NBT (0.05% [w/v] NBT, 50 mM Na_2_HPO_4_, 0.05% Tween 20, pH 7.8) solution overnight at 27 °C under light. After staining, the leaves were soaked in 95% ethanol overnight to remove chlorophyll.

### Catalase (CAT) Assay

For the CAT assay, the seedlings were transferred to basal medium supplemented with 0 or 150 mM NaCl after growing for 7 days in beakers. Twenty-four hours later, the aerial parts of the plants were excised and subjected to the analysis of CAT activity. An approximately 0.07-g sample of the aerial tissues was added to 1 mL of 50 mM sodium phosphate buffer (pH 6.8). After homogenization, the mixture was centrifuged at 12,000 × *g* for 20 min at 4 °C. After centrifugation, the supernatant was used as the source of enzymes. Then, 0.2 mL of the supernatant was added to 2.7 mL of 100 mM sodium phosphate buffer (pH 7.0) and 0.1 mL of 1 M H_2_O_2_. The decrease in the absorbance at 240 nm was recorded for 1 min. CAT activity was calculated using an extinction coefficient of 40 mM^−1^ cm^−1^ and was expressed as M H_2_O_2_ oxidized minutes^−1^ mg^−1^ protein (Scandalios et al. 1983).

### Measurement of Ion Contents

For the measurement of Na^+^ and K^+^ levels, the seedlings were transferred to basal medium supplemented with 0 or 150 mM NaCl after growing for 7 days in beakers. Ninety-six hours later, the aerial parts of the plants were excised, washed twice with ultrapure water, and subsequently vacuum dried overnight. The dried samples were digested using nitric and hydrochloric acids at a 4:1 ratio, boiled at 200 °C for 2 h, and subsequently filtered after 1 h of equilibration at room temperature. The levels of Na^+^ and K^+^ in the resulting filtrates were determined using an inductively coupled plasma optical emission spectrometer (ICP-OES, PerkinElmer Optima 8000).

### Microarray and Data Analysis

For the microarray experiment, the seedlings were transferred to basal medium supplemented with 0 or 150 mM NaCl after growing for 7 days in beakers. Twenty-four hours later, the aerial parts of the plant were subjected to RNA extraction according to the method described above. The DNase-treated total RNA was amplified and labeled with cyanine 3 (Cy3) using a Low-Input Quick Amp Labeling Kit, One-Color (Agilent, USA) following the manufacturer’s instructions. Labeled cRNA was fragmented by incubation at 60 °C for 30 min. After fragmentation, labeled cRNA was pooled and hybridized to the Agilent Rice Gene Expression 4 × 44 K Microarray (Agilent Technologies) as described by the manufacturer. The array image was analyzed with Feature Extraction software version 10.7.1.1 (Agilent Technologies) using the default setting. All microarray data are available at the Gene Expression Omnibus (GEO, http://www.ncbi.nlm.nih.gov/geo) under the accession number GSE160238. To identify DEGs, the raw microarray data were analyzed with the R/Bioconductor Limma package (Ritchie et al. 2015). The uploaded data were background-corrected using the ‘normexp’ method and normalized using the ‘quantile’ method. Each up- or downregulated DEP exhibited a log_2_-fold change > 1 or < 1, respectively, with a *P-value* < 0. 05 based on the FDR-corrected Student’s *t*-test. Each DEP-annotated gene is listed and described in the corresponding Additional file. Common DEG ontology graphical analysis was conducted using agriGO v2.0 online software (Tian et al. 2017).

## Supplementary Information

**Additional file 1.Figure S1** The number of transcription factors in the AP2/ERF, bHLH, WRKY, bZIP, MYB, NAC, C2H2, and other families in *Arabidopsis*, maize, *indica* rice, and *japonica* rice. The data were retrieved from the PlantTFDB v5.0 website (http://planttfdb.cbi.pku.edu.cn/). **Figure S2** Structural comparison of putative OsERF106-encoding transcripts between the Rice Annotation Project Database (RAP-DB, http://rapdb.dna.affrc.go.jp/) and the Michigan State University Rice Genome Annotation Project (MSU RGAP, http://rice.plantbiology.msu.edu/). The coding sequences of two Os08t0537900 transcripts (upper panel) and six Os08g42550 transcripts (lower panel) were documented on the RAP-DB and MSU RGAP websites, respectively. One typical AP2/EREBP domain (red bar) is present in the Os08g42550.1-encoded protein. The ScanProsite tool of ExPASy (http://www.expasy.org/) was used to retrieve the AP2/EREBP domain. **Figure S3** Os08t0537900-01 and Os08g42550.2 to Os08g42550.6 encode an O-fucosyltransferase (O-FucT) without the AP2/EREBP domain. The data were analyzed with NCBI SmartBLAST (http://blast.ncbi.nlm.nih.gov/smartblast/). **Figure S4** The coding sequence (CDS) of Os08t0537900-02 lacks an ATG-start codon. The image was taken from the Rice Annotation Project Database (RAP-DB, http://rapdb.dna.affrc.go.jp/). **Figure S5** Os08g42550.1 encodes an AP2/EREBP domain-containing O-fucosyltransferase (O-FucT)-like protein, which is homologous to AtO-FucT13 and GmO-FucT13 but not to any AtAP2/ERFs or GmAP2/ERFs. The data were analyzed with NCBI SmartBLAST (http://blast.ncbi.nlm.nih.gov/smartblast/). **Figure S6** Gene structure and amino acid sequence alignment of OsERF106MZ (GenBank accession No. MZ561461). (A) Structure of *OsERF106MZ*. (B) Amino acid sequence alignment of OsERF105, OsERF106MZ, and OsERF107 together with their homologs, AtERF108 and AtERF113. The predicted AP2/EREBP domain is underlined in green. The conserved alanine 14 and aspartic acid 19 residues of the AP2/EREBP domain are indicated with red and blue asterisks, respectively. **Figure S7** Phylogenetic analysis of OsERF105, OsERF106MZ, and OsERF107 together with AtERF108, AtERF113, AtO-FucT13, and GmO-FucT13 using the neighbor-joining method. Numbers next to the descendants indicate confidence values based on the bootstrap method. **Figure S8** The subcellular localization of OsERF106MZ-GFP and GFP-OsERF106MZ in *Oncidium* ‘Sweet Sugar’ suspension cells. **Figure S9** Characterization of a retrotransposon insertion *Oserf106mz* mutant line, H0159. (A) A schematic diagram of the retrotransposon insertion site (indicated with a red triangle) in Os08g42550.1 and the *OsERF106MZ* gene. (B) Identification of homozygous H0159 lines by genomic DNA genotyping. The Arabic numerals represent the individual rice plants within each genotype. (C) Quantification of Os08g42550.s mRNA levels in Hitomebore and *Oserf106mz* plants by qPCR. The values are the mean ± SE of five biological replicates, each with two technical replicates. The positions of the primers used for genotyping (B) and qPCR (C) are indicated by green and blue arrows, respectively, in (A). The primer sequences are listed in Additional file 2: Table S1. The seedlings were grown on basal medium for 11 days and then subjected to genotyping and qPCR assays. **Figure S10** MDA (left panel) and Na^+^ (right panel) levels in the roots of Tainung 67 and *OsERF106MZ*-overexpressing rice plants. **Figure S11** GO analysis of the common DEGs according to biological processes and molecular functions. **Figure S12** Comparison between the coding sequence of Os08t0537900-02 and the cDNA sequence of *OsERF106MZ* gene from both the TNG67 and Hitomebore (Hito) backgrounds. The 5′ UTR, exon 1, exon 2, and 3′ UTR within the cDNA sequence of *OsERF106MZ* gene are underlined in green, red, blue, and purple, respectively. The ATG-start and TAA-stop codons of *OsERF106MZ* gene are indicated by yellow boxes. **Figure S13** Nuclear localization signal (NLS) prediction in OsERF106MZ as well as its homologs OsERF105 and OsERF107. The data were analyzed with NLS Mapper (http://nls-mapper.iab.keio.ac.jp/). OsbHLH068 is a nuclear-localized protein that has been documented in a previous study (Chen et al. 2017) and is used as a positive control. **Figure S14** Nucleotide sequence alignment of *OsERF105* (Os05g36100), *OsERF106* (Os08g42550.1), and *OsERF107* (Os02g32140), three genes belonging to the rice ERF-Xc subgroup, from the region encoding the AP2/EREBP domain (underlined in green) to the translation stop site. The positions of the *OsERF106* gene-specific primers (GSPs) used in 5′ and 3′ RACE experiments are underlined in red and blue, respectively.

**Additional file 2. Table S1** Primers used in this study.

**Additional file 3. Data S1** OsERF106-related DEGs.

**Additional file 4. Data S2** Common DEGs.

## Data Availability

All data supporting the conclusions of this article are provided within the article and its additional files.

## Abbreviations

ABA: Abscisic acid
aa: Amino acid
AP2/EREBP: APETALA2/ethylene-responsive element binding protein
CAT: Catalase
CDS: Coding DNA sequence
CRE: *cis*-Regulatory (or *cis*-acting) element
CRT/DRE: C-repeat/dehydration-responsive element
DAB: 3,3′-Diaminobenzidine
DBD: DNA-binding domain
GFP: Green fluorescent protein
GSPs: Gene-specific primers
GUS: β-Glucuronidase
ICP-OES: Inductively coupled plasma-optical emission spectrometry
MDA: Malondialdehyde
MSU RGAP: Michigan State University Rice Genome Annotation Project Database
NBT: Nitro blue tetrazolium
*O*-FucT: *O*-Fucosyltransferase
ORF: Open reading frame
qPCR: Quantitative PCR
RACE-PCR: Rapid amplification of cDNA ends-PCR
RAP-DB: Rice Annotation Project Database
ROS: Reactive oxygen species
RT-PCR: Reverse transcription-PCR
TFs: Transcription factors
TNG67: Tainung 67 (*Oryza sativa* L. spp.* japonica* cv. Tainung 67)
UTR: Untranslated region
