# Supplementary material for: Overexpression of a Novel ERF-X-Type Transcription Factor, OsERF106MZ, Reduces Shoot Growth and Tolerance to Salinity Stress in Rice
Source: Rice (N Y). 2021 Sep 20;14:82. doi: 10.1186/s12284-021-00525-5 (PMC8452809; doi:10.1186/s12284-021-00525-5)
Supplement: Supplementary file 1 — Additional file 1.Figure S1 The number of transcription factors in the AP2/ERF, bHLH, WRKY, bZIP, MYB, NAC, C2H2, and other families in Arabidopsis, maize, indica rice, and japonica rice. The data were retrieved from the PlantTFDB v5.0 website (http://planttfdb.cbi.pku.edu.cn/). Figure S2 Structural comparison of putative OsERF106-encoding transcripts between the Rice Annotation Project Database (RAP-DB, http://rapdb.dna.affrc.go.jp/) and the Michigan State University Rice Genome Annotation Project (MSU RGAP, http://rice.plantbiology.msu.edu/). The coding sequences of two Os08t0537900 transcripts (upper panel) and six Os08g42550 transcripts (lower panel) were documented on the RAP-DB and MSU RGAP websites, respectively. One typical AP2/EREBP domain (red bar) is present in the Os08g42550.1-encoded protein. The ScanProsite tool of ExPASy (http://www.expasy.org/) was used to retrieve the AP2/EREBP domain. Figure S3 Os08t0537900-01 and Os08g42550.2 to Os08g42550.6 encode an O-fucosyltransferase (O-FucT) without the AP2/EREBP domain. The data were analyzed with NCBI SmartBLAST (http://blast.ncbi.nlm.nih.gov/smartblast/). Figure S4 The coding sequence (CDS) of Os08t0537900-02 lacks an ATG-start codon. The image was taken from the Rice Annotation Project Database (RAP-DB, http://rapdb.dna.affrc.go.jp/). Figure S5 Os08g42550.1 encodes an AP2/EREBP domain-containing O-fucosyltransferase (O-FucT)-like protein, which is homologous to AtO-FucT13 and GmO-FucT13 but not to any AtAP2/ERFs or GmAP2/ERFs. The data were analyzed with NCBI SmartBLAST (http://blast.ncbi.nlm.nih.gov/smartblast/). Figure S6 Gene structure and amino acid sequence alignment of OsERF106MZ (GenBank accession No. MZ561461). (A) Structure of OsERF106MZ. (B) Amino acid sequence alignment of OsERF105, OsERF106MZ, and OsERF107 together with their homologs, AtERF108 and AtERF113. The predicted AP2/EREBP domain is underlined in green. The conserved alanine 14 and aspartic acid 19 residues of the AP2/EREBP domain ar [file 12284_2021_525_MOESM1_ESM.pdf]

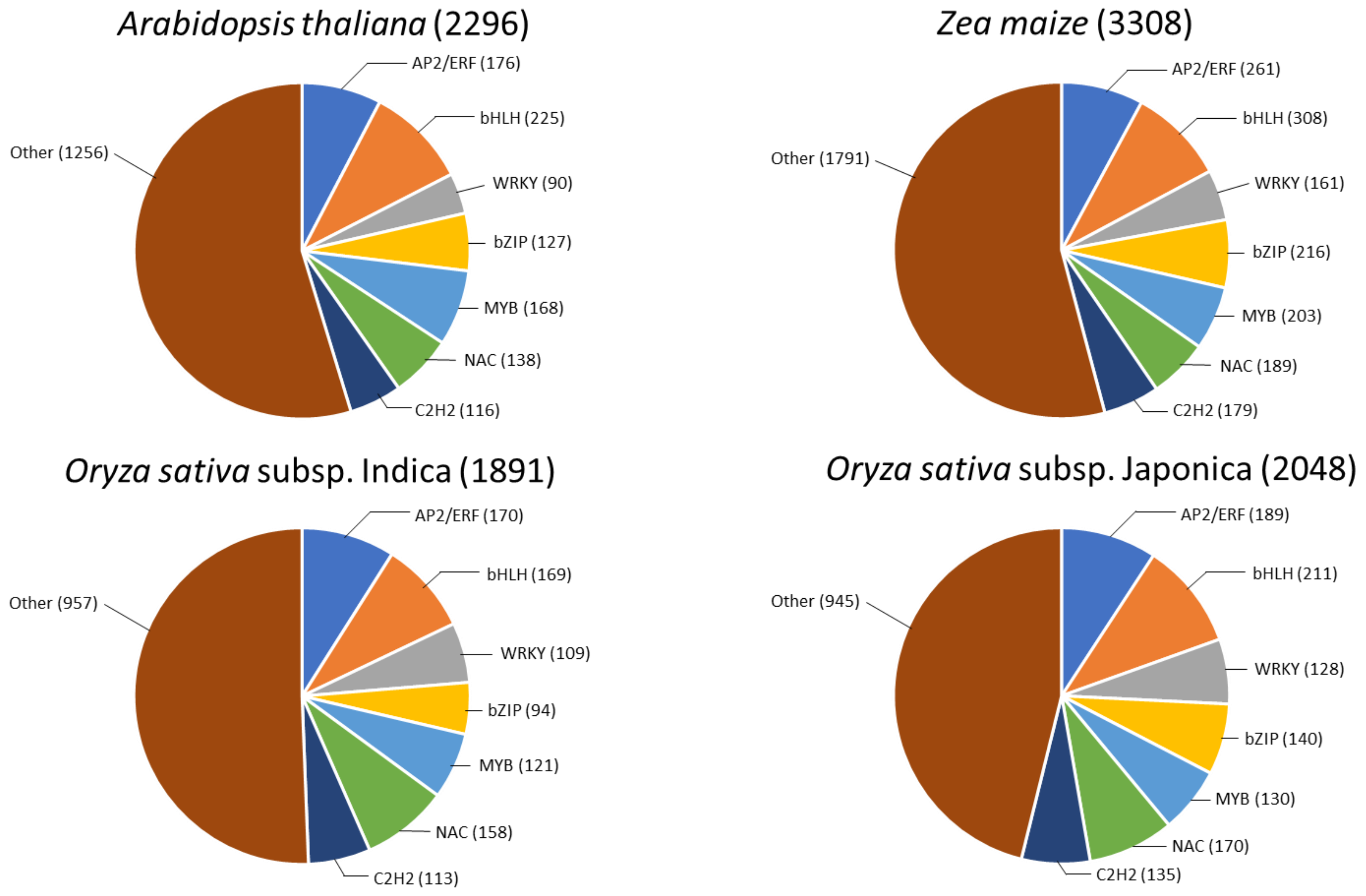

Figure S1 The number of transcription factors in the AP2/ERF, bHLH, WRKY, bZIP, MYB, NAC, C2H2, and other families in *Arabidopsis*, maize, *indica* rice, and *japonica* rice. The data were retrieved from the PlantTFDB v5.0 website (http://planttfdb.cbi.pku.edu.cn/).

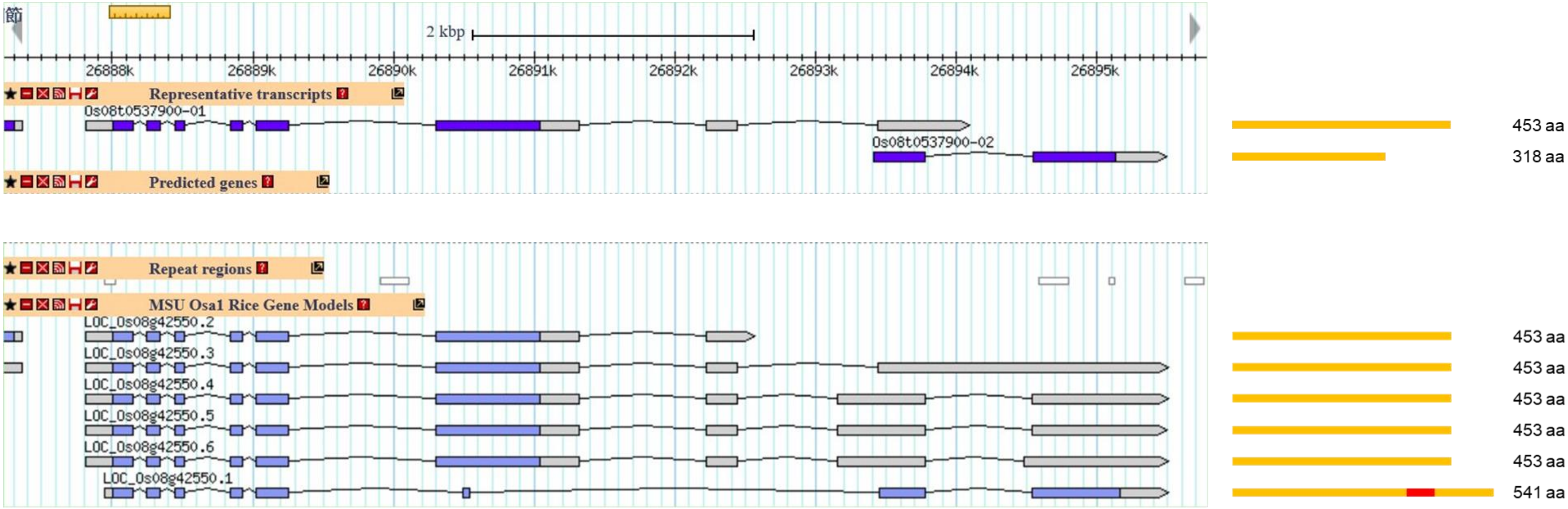

Figure S2 Structural comparison of putative OsERF106-encoding transcripts between the Rice Annotation Project Database (RAP-DB, http://rapdb.dna.affrc.go.jp/) and the Michigan State University Rice Genome Annotation Project (MSU RGAP, http://rice.plantbiology.msu.edu/). The coding sequences of two Os08t0537900 transcripts (upper panel) and six Os08g42550 transcripts (lower panel) were documented on the RAP-DB and MSU RGAP websites, respectively. One typical AP2/EREBP domain (red bar) is present in the Os08g42550.1-encoded protein. The ScanProsite tool of ExPASy (http://www.expasy.org/) was used to retrieve the AP2/EREBP domain.

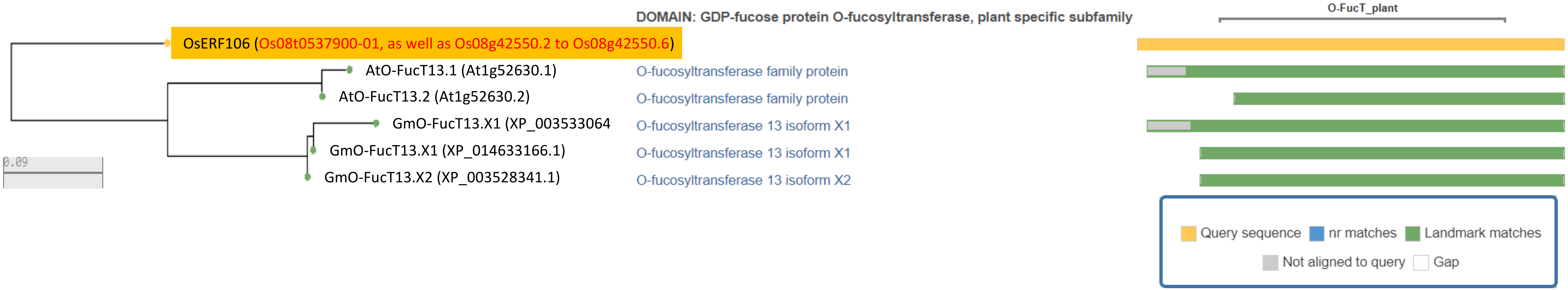

Figure S3 Os08t0537900-01 and Os08g42550.2 to Os08g42550.6 encode an O-fucosyltransferase (O-FucT) without the AP2/EREBP domain. The data were analyzed with NCBI SmartBLAST (http://blast.ncbi.nlm.nih.gov/smartblast/).

CDS

957 bp

```
>Os08t0537900-02 Hypothetical conserved gene.
ATCAGTTACTCCTCTTACCAGTGCAGCTCTGCATCGTCAGCGATCAAGGGTACCAGGGTG
GATCGCCTACGAGAAGCTAGTGACACAGGGCATCATCGTGGTCGGCACAAGAGGGCCCGC
TCCTCTCCTCCACGCCATGACGACGAGCCGCCACCGCCGCCGCCGCTGTCTCCGGCGGTG
ACAGGAGGAGCACACGGCGGCGCCGCGTGGCACTCAACGGTGCTCGCGGAGGACGTCGAA
AGTGCCGTCATCGTCGCGGCGCTGACGCACGTCATCAGCTCCACGGCGGCGGAGGTGACC
ACGGCCGTTCCTCCGGTGACGGTTGCGCCGCAGCGAGCTGCCACGGCTACCATGTTCGGA
CAGCAAGCCGCCGCGCGGCTTTCCTCCTCTGCCGTCGTCGAGCGGATCAGCagcgacggc
gccggagcagcagcagccacggcggcgctaccgcggcgtgcggcagcggccgtgggggaa
gtgggcggcggagatccgcgacccggtgaaggcggcgcgggtgtggctcggcaccttcga
caccgccgaggacgccgcccgcgcctacgacgccgccgccgtccgcttcaagggctccaa
ggccaaggtcaacttcccCGACGAAGTCGCCGGCGCCAGCATCGCCGCCGTCCAGCTGCC
ACGCCATCATCAACACCACCCCCCAACGTCACTGCCGCCGCTGCCGGTGCCGGCGCATCT
GAGGCCACACTTCAGCCTCGCCGGCGGCCAGAGCACCACACCGGCGCCCGCAGCCGCCGC
CGTCGCCGCTCCGCCGCGGGAGGAGTTCCCCGACCTCAGCAGCTACGCGCACATACTGCA
GAGCGGCGACCTGGAGTACGACTTCCACGCCGCGGTTTCTGCCGGACTAACGACGACAGC
TGGGCGAtcgtcgtcgtcgtcgtcattgtcgatgccgccgccatccgagGATCTTGA
```

Figure S4 The coding sequence (CDS) of Os08t0537900-02 lacks an ATG-start codon. The image was taken from the Rice Annotation Project Database (RAP-DB, http://rapdb.dna.affrc.go.jp/).

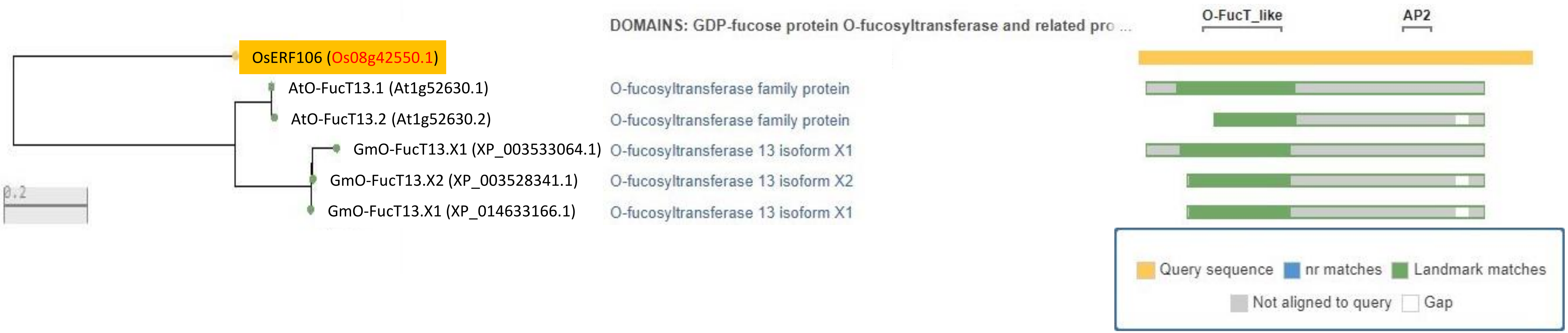

Figure S5 Os08g42550.1 encodes an AP2/EREBP domain-containing O-fucosyltransferase (O-FucT)-like protein, which is homologous to AtO-FucT13 and GmO-FucT13 but not to any AtAP2/ERFs or GmAP2/ERFs. The data were analyzed with NCBI SmartBLAST (http://blast.ncbi.nlm.nih.gov/smartblast/).

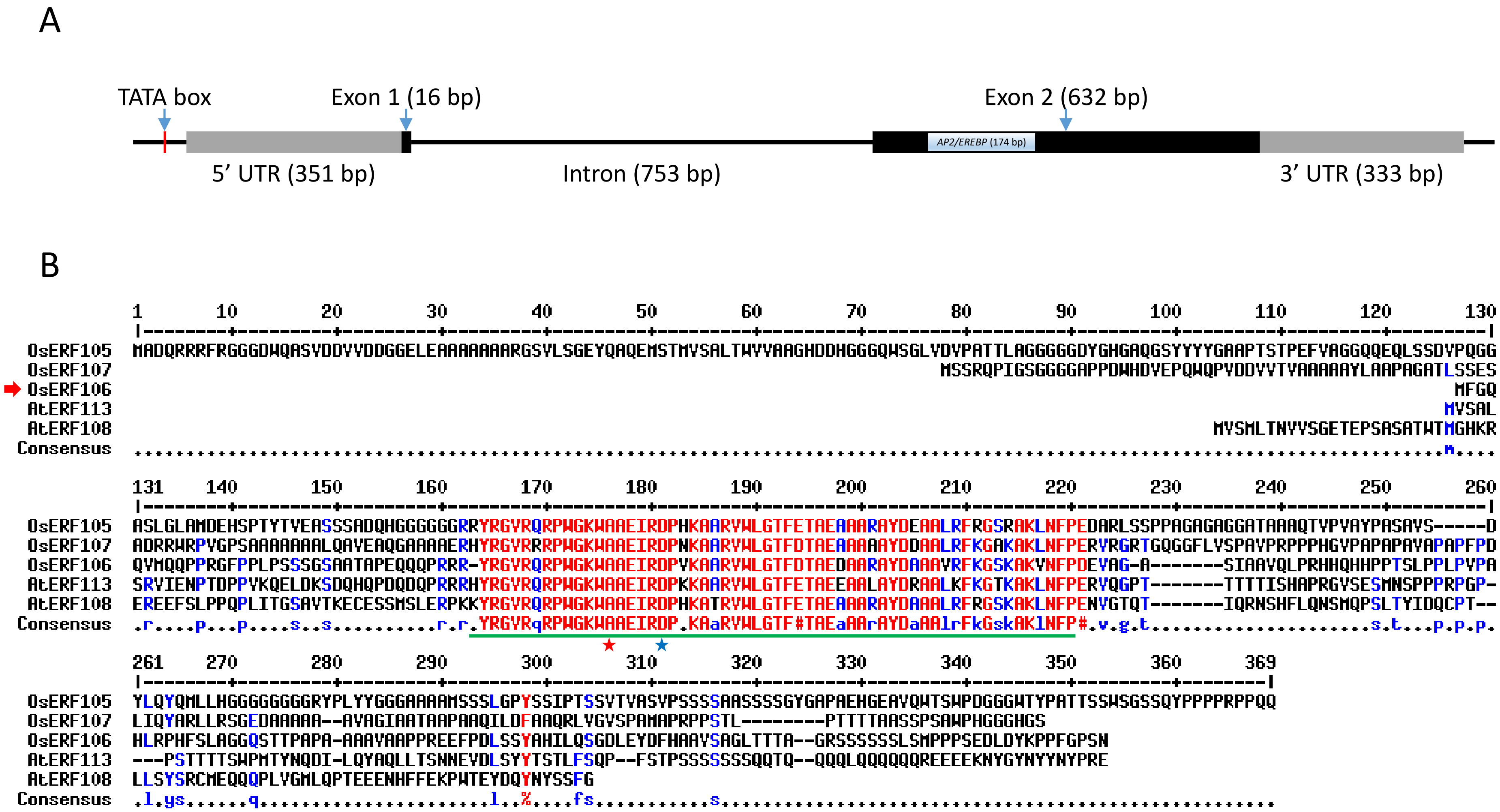

Figure S6 Gene structure and amino acid sequence alignment of OsERF106MZ (GenBank accession No. MZ561461). (A) Structure of *OsERF106MZ*. (B) Amino acid sequence alignment of OsERF105, OsERF106MZ, and OsERF107 together with their homologs, AtERF108 and AtERF113. The predicted AP2/EREBP domain is underlined in green. The conserved alanine 14 and aspartic acid 19 residues of the AP2/EREBP domain are indicated with red and blue asterisks, respectively.

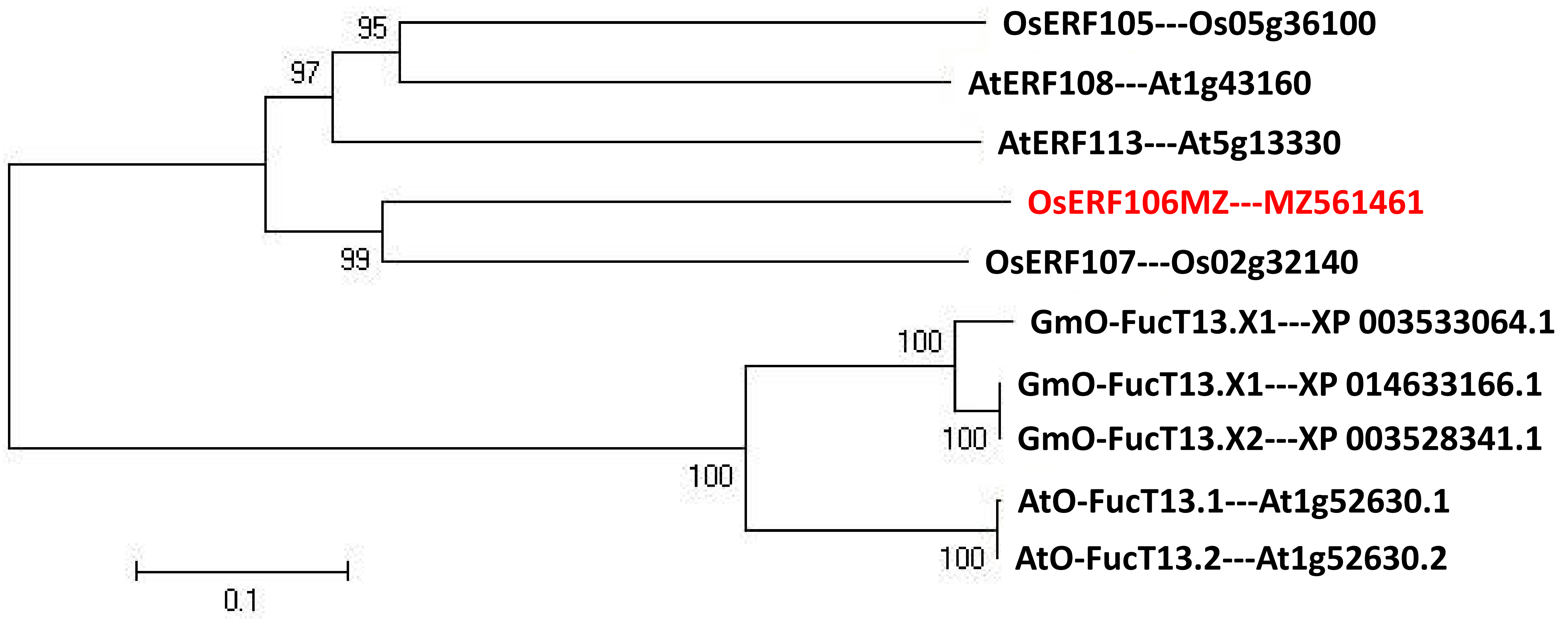

Figure S7 Phylogenetic analysis of OsERF105, OsERF106MZ, and OsERF107 together with AtERF108, AtERF113, AtO-FucT13, and GmO-FucT13 using the neighbor-joining method. Numbers next to the descendants indicate confidence values based on the bootstrap method.

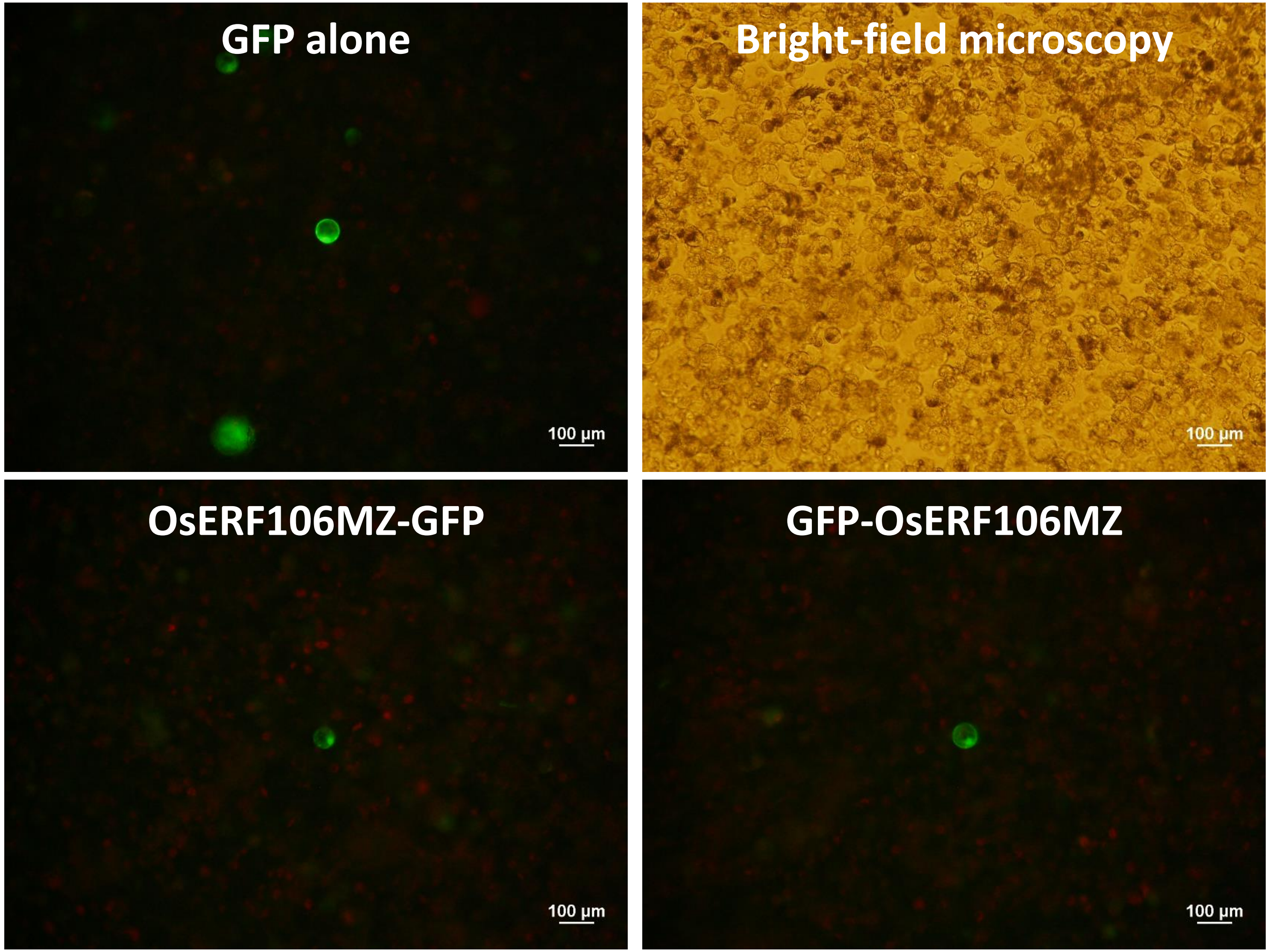

Figure S8 The subcellular localization of OsERF106MZ-GFP and GFP-OsERF106MZ in *Oncidium* ‘Sweet Sugar’ suspension cells.

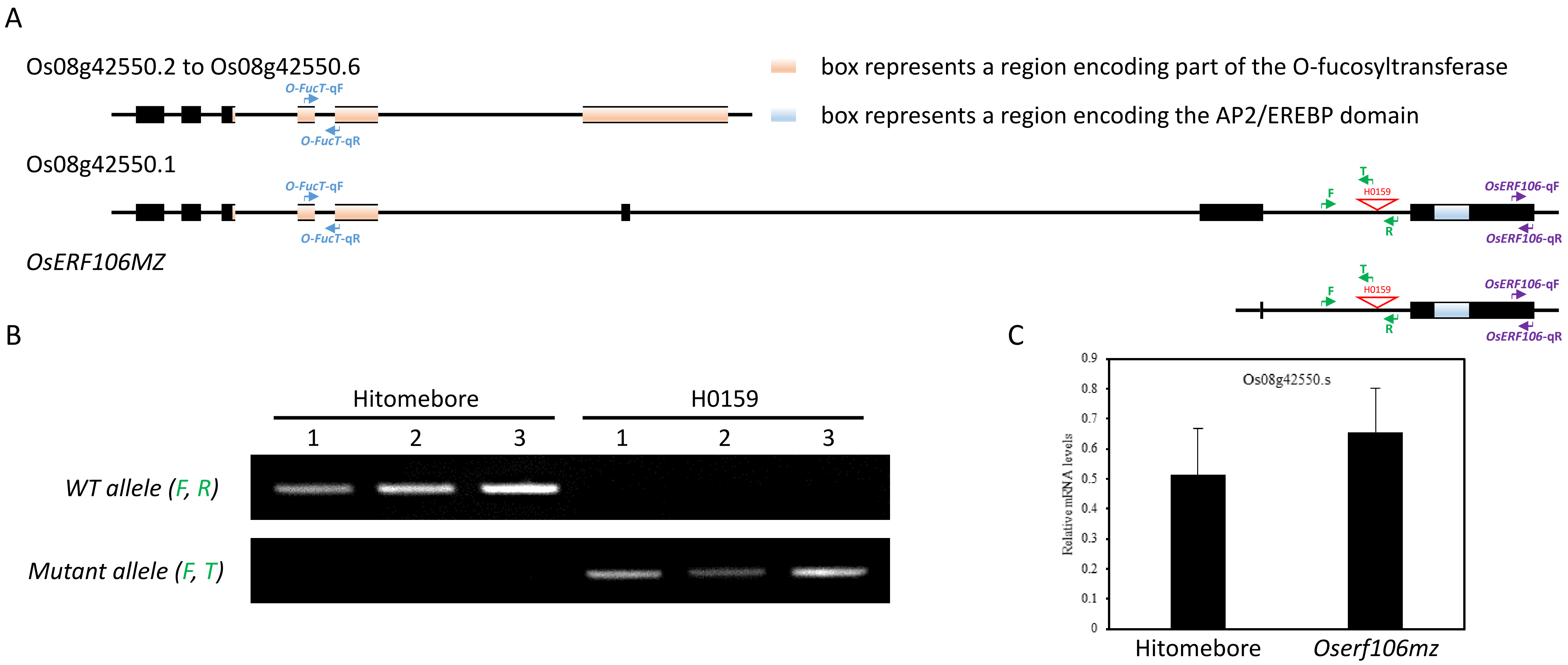

Figure S9 Characterization of a retrotransposon insertion *Oserf106mz* mutant line, H0159. (A) A schematic diagram of the retrotransposon insertion site (indicated with a red triangle) in Os08g42550.1 and the *OsERF106MZ* gene. (B) Identification of homozygous H0159 lines by genomic DNA genotyping. The Arabic numerals represent the individual rice plants within each genotype. (C) Quantification of Os08g42550.s mRNA levels in Hitomebore and *Oserf106mz* plants by qPCR. The values are the mean ± SE of five biological replicates, each with two technical replicates. The positions of the primers used for genotyping (B) and qPCR (C) are indicated by green and blue arrows, respectively, in (A). The primer sequences are listed in Additional file 2: Table S1. The seedlings were grown on basal medium for 11 days and then subjected to genotyping and qPCR assays.

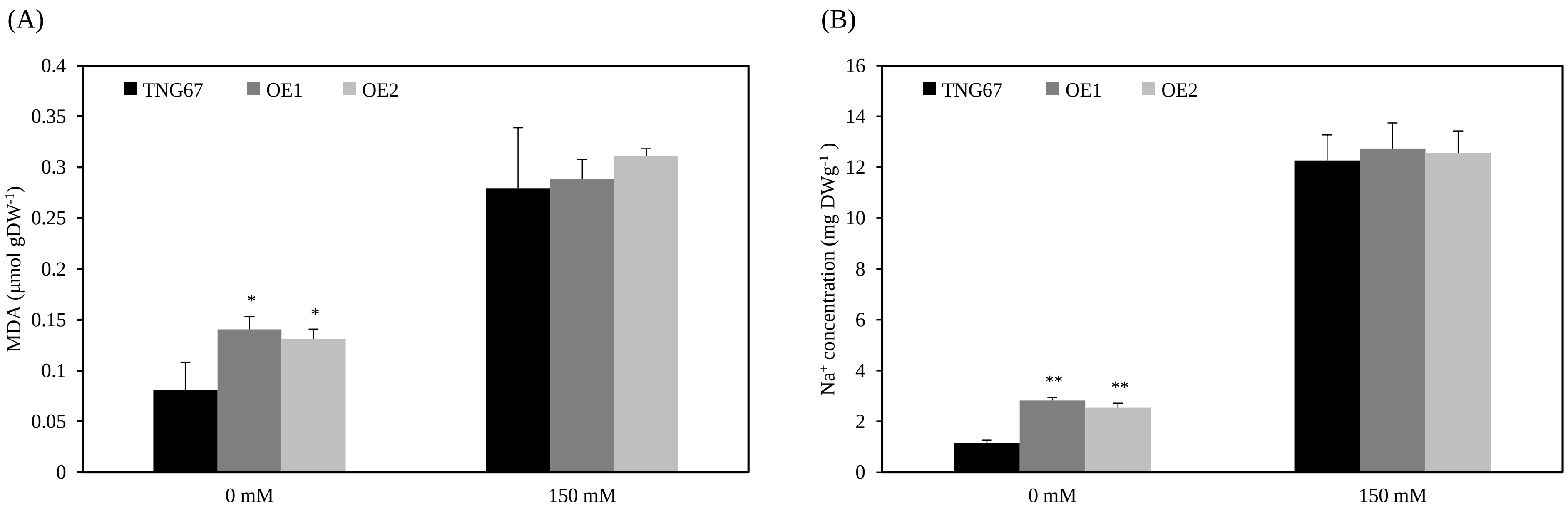

Figure S10 MDA (left panel) and Na^{+} (right panel) contents in the roots of Tainung 67 and OsERF106MZ-overexpressing rice plants.

Biological process

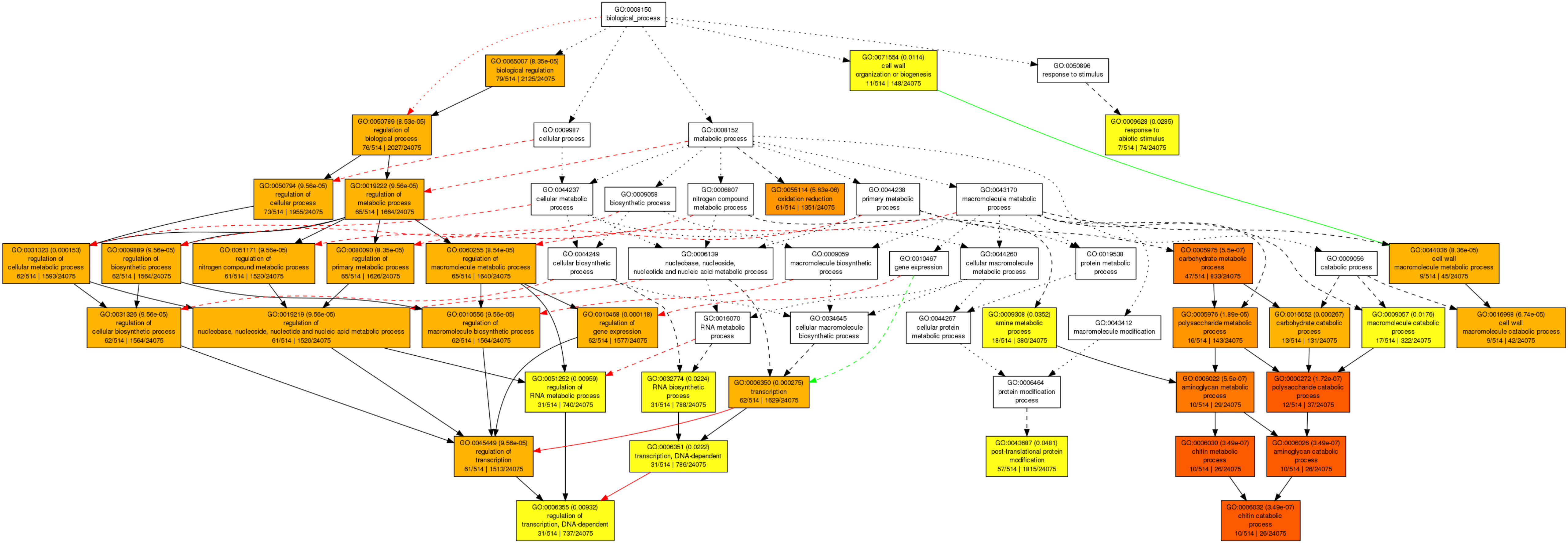

Figure S11 GO analysis of the common DEGs according to biological processes and molecular functions.

continued

Molecular function

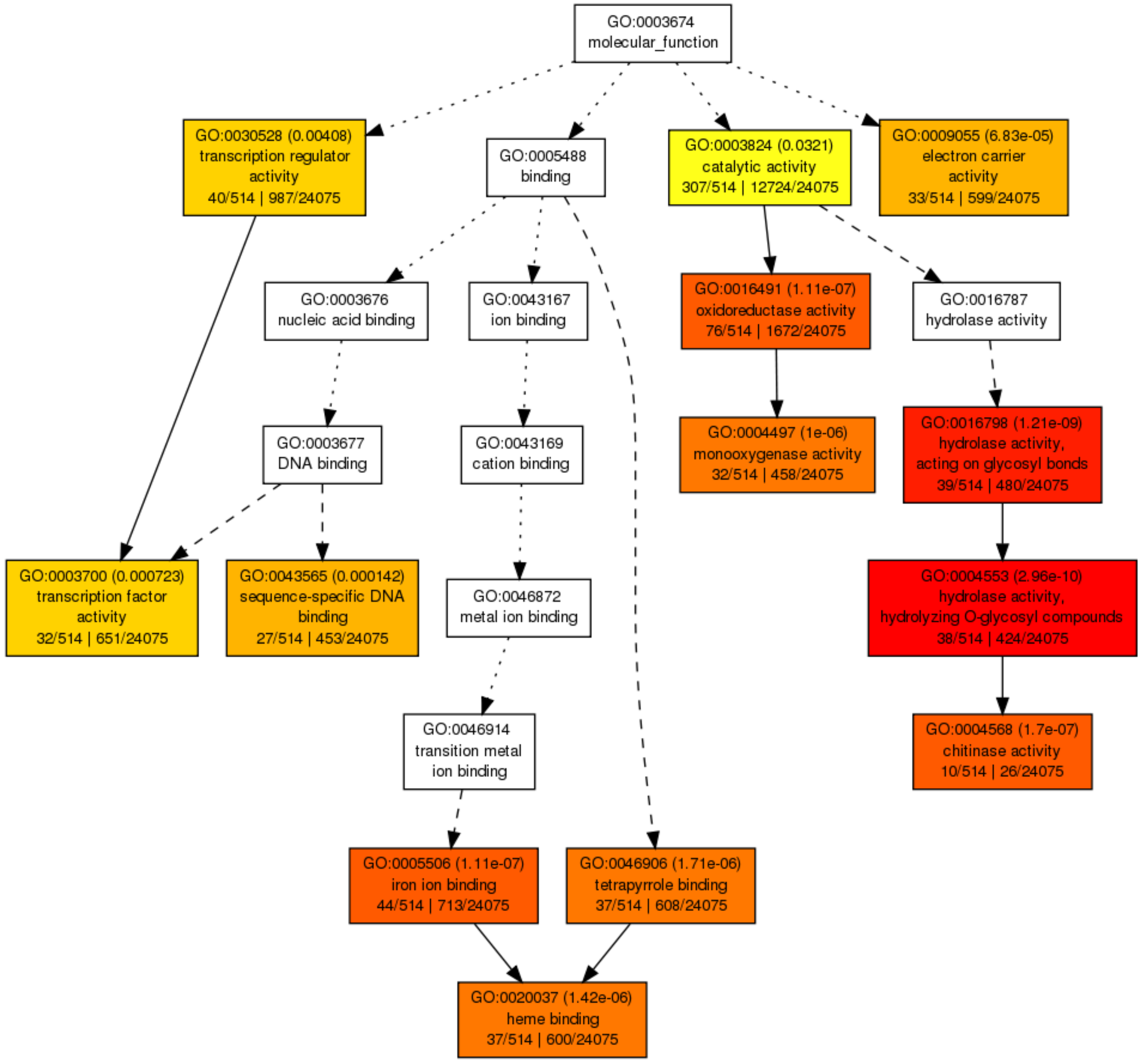

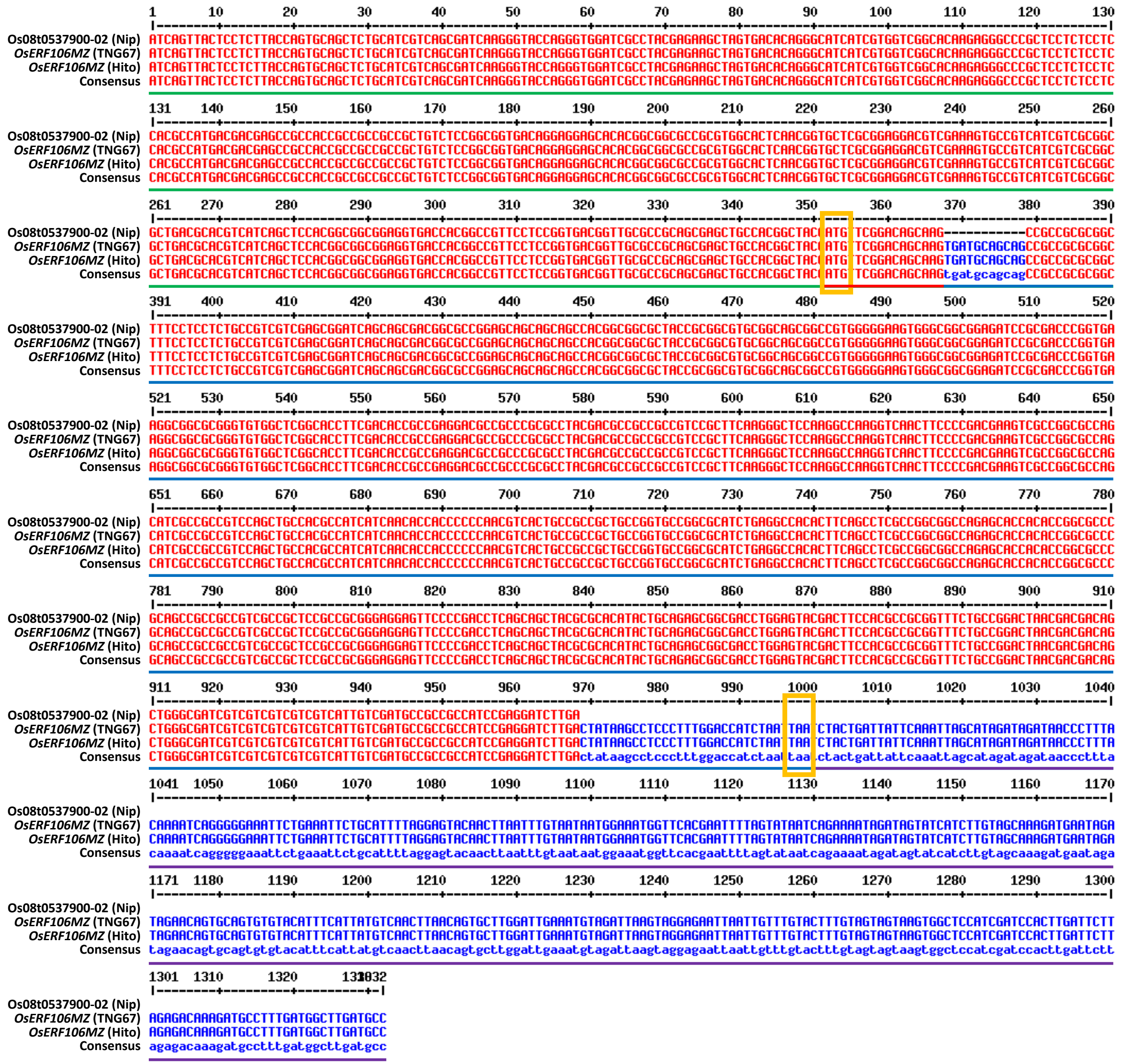

Figure S12 Comparison between the coding sequence of Os08t0537900-02 and the cDNA sequence of *OsERF106MZ* gene from both the TNG67 and Hitomebore (Hito) backgrounds. The 5’ UTR, exon 1, exon 2, and 3’ UTR within the cDNA sequence of *OsERF106MZ* gene are underlined in green, red, blue, and purple, respectively. The ATG-start and TAA-stop codons of *OsERF106MZ* gene are indicated by yellow boxes.

OsERF105

cNLS Mapper Result

Predicted NLSs in query sequence

```
MADQRRRFRGGGDWQASVDDVVDDGGELEAAAAAAARGSVLSGEYQAQEM  50
STMVSALTWVVAAGHDDHGGGQWSGLVDVPATTLAGGGGGDYGHGAQGSY 100
YYYGAAPTSTPEFVAGGQQEQLSSDVPQGGASLGLAMDEHSPTYTVEASS 150
SADQHGGGGGGRRYRGVRQRPWGKWAAEIRDPHKAARVWLGTFETAEAAA 200
RAYDEAALRFRGSRAKLNFPEDARLSSPPAGAGAGGATAAAQTVPVAYPA 250
SAVSDYLQYQMLLHGGGGGGGRYPLYYGGGAAAAMSSSLGPYSSIPTSS 300
VTVASVPSSSSAASSSSGYGAPAEHGEAVQWTSWPDGGGWTYPATTSSWS 350
GSSQYPPPPRPPQQ                                     364
```

Predicted monopartite NLS

Pos.	Sequence	Score

Predicted bipartite NLS

Pos.	Sequence	Score

OsERF106MZ

cNLS Mapper Result

Predicted NLSs in query sequence

```
MFGQQVMQQPPRGFPPLPSSSGSAATAPEQQQPRRRYRGVRQRPWGKWAA  50
EIRDPVKAARVWLGTFDTAEDAARAYDAAAVRFKGSKAKVNFPDEVAGAS 100
IAAVQLPRHHQHHPPTSLPPLPVPAHLRPHFSLAGGQSTTPAPAAAAVAA 150
PPREEFPDLSSYAHILQSGDLEYDFHAAVSAGLTTTAGRSSSSSSLSMPP 200
PSEDLDYKPPFGPSN                                    215
```

Predicted monopartite NLS

Pos.	Sequence	Score

Predicted bipartite NLS

Pos.	Sequence	Score

OsERF107

cNLS Mapper Result

Predicted NLSs in query sequence

```
MSSRQPIGSGGGGAPPDWHDVEPQWQPVDDVVTVAAAAAYLAAPAGATLS  50
SESADRRWRPVGPSAAAAAAALQAVEAQGAAAAERHYRGVRRRPWGKWAA 100
EIRDPNKAARVWLGTFDTAEAAAAAYDDAALRFKGAKAKLNFPERVRGRT 150
GQGGFLVSPAVPRPPPHGVPAPAPAVAPAPFPDLIQYARLLRSGEDAAAA 200
AAVAGIAATAAPAAQILDFAAQRLVGVSPAMAPRPPSTLPTTTTAASSPS 250
AWPHGGGHGS                                         260
```

Predicted monopartite NLS

Pos.	Sequence	Score

Predicted bipartite NLS

Pos.	Sequence	Score

OsbHLH068

cNLS Mapper Result

Predicted NLSs in query sequence

```
MGDHQMMHAAPAAMYNGGGGTTSSHGVWWSNAVGVPAAATCSTTTELAGY  50
TAWSSALAAGYDGMVADNGGKQAKSTTTASSESPGNNSSVTFQEPASIPD 100
PAAVAAVPQPGLAGFTDWTQPFMNNGAGLHEFLQDGHHDMSASSLMNHSS 150
NNLALQQAGHHHELLSSFGSDLLLSPTSPYGGFQSSLLRSLMEPTAKQQQ 200
QQPALAGLQQYHQYQQQMGHAPAAAAKFAQAVGARDSLQFTNDAPFWNPS 250
AGFGMPAAVAVAAAAAQDQASVRSAKRSSPAPPRAAATLALKTAMEGVGD 300
SSSVITKKETAFKKPRLETPSPLPTFKVRKEKLGDRITALQQLVSPFGKT 350
DTASVLHETIEYIKFLHDQVGALSAPYLKNGAHQVPHLKNSSPDKSKHGE 400
ISLKGRGLCLVPISSTFAVASEVPVELWTPFGANFIR              437
```

Predicted monopartite NLS

Pos.	Sequence	Score
310	TAFKKPRLE	7.5

Predicted bipartite NLS

Pos.	Sequence	Score

Figure S13 Nuclear localization signal (NLS) prediction in OsERF106MZ as well as its homologs OsERF105 and OsERF107. The data were analyzed with NLS Mapper (http://nls-mapper.iab.keio.ac.jp/). OsbHLH068 is a nuclear-localized protein that has been documented in a previous study (Chen et al. 2017) and is used as a positive control.

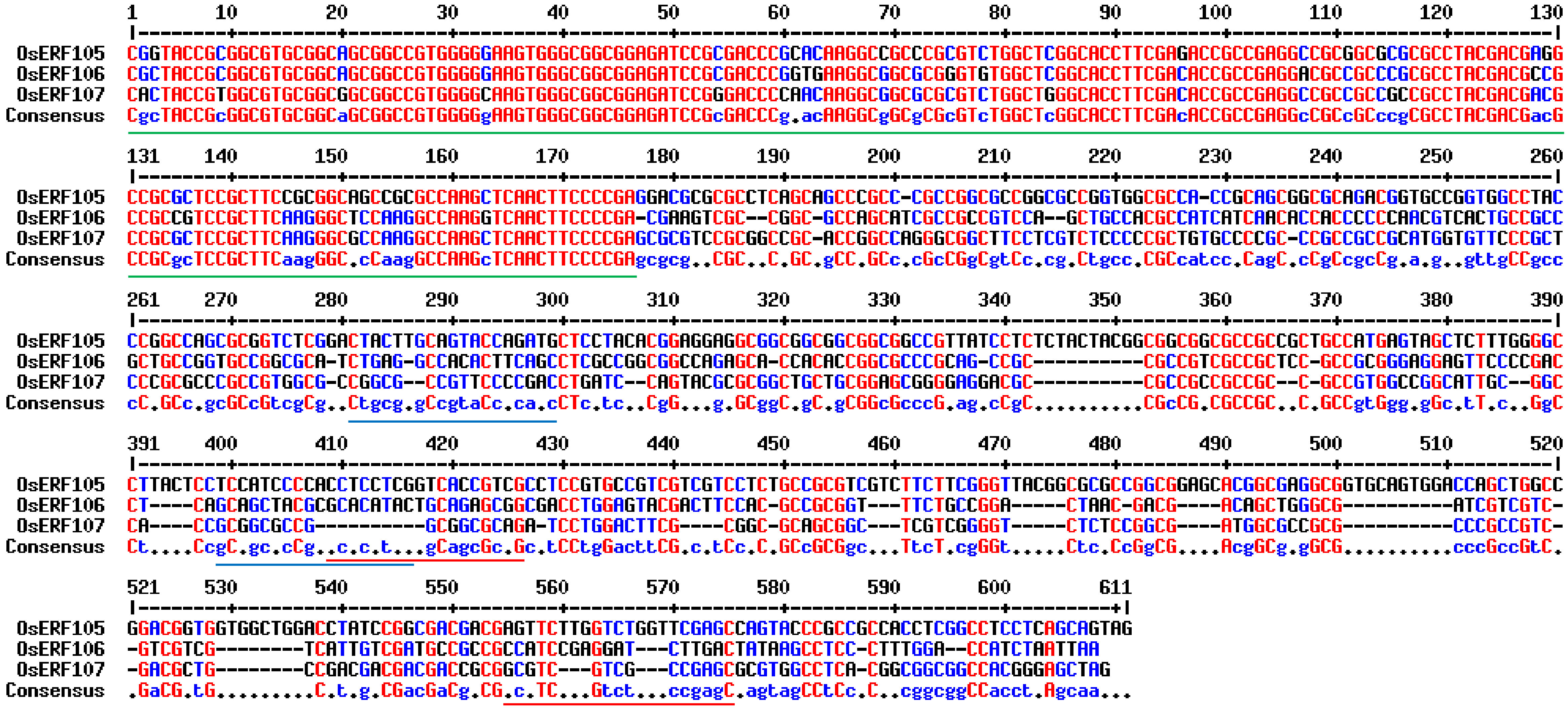

Figure S14 Nucleotide sequence alignment of *OsERF105* (Os05g36100), *OsERF106* (Os08g42550.1), and *OsERF107* (Os02g32140), three genes belonging to the rice ERF-Xc subgroup, from the region encoding the AP2/EREBP domain (underlined in green) to the translation stop site. The positions of the *OsERF106* gene-specific primers (GSPs) used in 5' and 3' RACE experiments are underlined in red and blue, respectively.
