# Supplementary material for: Overexpression of a Novel ERF-X-Type Transcription Factor, OsERF106MZ, Reduces Shoot Growth and Tolerance to Salinity Stress in Rice
Source: Rice (N Y). 2021 Sep 20;14:82. doi: 10.1186/s12284-021-00525-5 (PMC8452809; doi:10.1186/s12284-021-00525-5)
Supplement: Supplementary file 2 — Additional file 2. Table S1 Primers used in this study. [file 12284_2021_525_MOESM2_ESM.pdf]

**Table S1** Primers used in this study.

| PCR Type | Gene | Sequence (5'-3') | Annotation |
|---|---|---|---|
| 5' RACE | *OsERF106* Os08g42550 | GTCAAGATCCTCGGATGG | 5' GSP |
| | | CCGCTCTGCAGTATGTGC | 5' Nested GSP |
| 3' RACE | *OsERF106* Os08g42550 | CTGAGGCCACACTTCAGC | 3' GSP |
| | | GCAGCTACGCGCACATAC | 3' Nested GSP |
| gDNA genotyping | *OsERF106* Os08g42550 | TCAACTTTGGGACAAAAGGG (F) CTGATACCATCTTAACTAACTTGC (T) CACACGGCAAAATTGAGGTA (R) | 397 bp (F, R) 428 bp (F, T) |
| qPCR | *OsERF106* Os08g42550 | GGTTTCTGCCGGACTAACGA (*OsERF106*-qF) GATGGTCCAAAGGGAGGCTT (*OsERF106*-qR) | 108 bp |
| | *O-FucT* | GCAACAATGGTTCTGCCCAA (*O-FucT*-qF) TCGAAAACGTCTGCAAATCCA (*O-FucT*-qG) | 72 bp |
| | *OsSOS1* Os12g44360 | CTCCGTGCTCATAGAATCGC ATACTCACTCAAGTGGGTCAATACC | 207 bp |
| | *OsHKT1.1* Os04g51820 | ATTAGCAGAGCACTGTGGAGGAA CCGACGAACCCGTAGGAAG | 245 bp |
| | *OsHKT1.3* Os02g07830 | CAGTTCATCTACCAAAACAATCCA AATACCTCACCACCAATCAGCA | 251 bp |
| | *OsAKT1* Os01g45990 | TACGACCGCCGATACAGAA CCAAATAAGCCACAAAGAAGG | 184 bp |

| | | | |
|---|---|---|---|
| | *OsABI5* | TCACAGGAGCGTGACCAAAG | 128 bp |
| | Os01g64000 | CCTGGCTCACACAAGGAGTT | |
| | *OsHOK24* | TACGAGTACGACCATCACCTAGACTAC | 90 bp |
| | Os02g43330 | TGGCCATATCTCCCACAGATC | |
| | *OsMAIF1* | GAAGCGGGATTGCGTCTACT | 104 bp |
| | Os02g44990 | TATCGACCTCTCCGGTCTCC | |
| | *OsSRO1c* | TCCCTATGCTTCTGACGGAGAT | 76 bp |
| | Os01g45990 | CAGTTGTACGTCCTCTGCAAAGTC | |
| | *OsNAC006* | CGAGGAGGTTGGTATAAGTGGTGA | 170 bp |
| | Os01g09550 | TATTTGATACAGGGTTCGTCGGC | |
| | *OsActin1* | CTCAGCACATTCCAGCAGATGTG | 126 bp |
| | Os03g50885 | GATAACAGCTCCTCTTGGCTTAGC | |
